# Synthesis and Biological Evaluation of a New Structural Simplified Analogue of cADPR, a Calcium-Mobilizing Secondary Messenger Firstly Isolated from Sea Urchin Eggs

**DOI:** 10.3390/md16030089

**Published:** 2018-03-10

**Authors:** Stefano D’Errico, Nicola Borbone, Bruno Catalanotti, Agnese Secondo, Tiziana Petrozziello, Ilaria Piccialli, Anna Pannaccione, Valeria Costantino, Luciano Mayol, Gennaro Piccialli, Giorgia Oliviero

**Affiliations:** 1 Dipartimento di Farmacia, Università degli Studi di Napoli Federico II, via Domenico Montesano 49, 80131 Napoli, Italy; stefano.derrico@unina.it (S.D.); nicola.borbone@unina.it (N.B.); bruno.catalanotti@unina.it (B.C.); valeria.costantino@unina.it (V.C.); mayoll@unina.it (L.M.); picciall@unina.it (G.P.); 2 SYSBIO, Centre of Systems Biology, University of Milano-Bicocca, Piazza della Scienza 2, 20126 Milano, Italy; 3 Divisione di Farmacologia, Dipartimento di Neuroscienze, Scienze Riproduttive e Odontostomatologiche, Università degli Studi di Napoli Federico II, Via Sergio Pansini 5, 80131 Napoli, Italy; agnese.secondo@unina.it (A.S.); tiziana.petrozziello@unina.it (T.P.); ilaria.piccialli@unina.it (I.P.); pannacio@unina.it (A.P.); 4 Dipartimento di Biologia Molecolare e Biotecnologie Mediche, Università degli Studi di Napoli Federico II, via Sergio Pansini 5, 80131 Napoli, Italy

**Keywords:** cADPR, calcium mobilization, PC12 neuronal cells, cyclization, nucleotides, macrocycle conformational sampling

## Abstract

Herein, we reported on the synthesis of cpIPP, which is a new structurally-reduced analogue of cyclic ADP-ribose (cADPR), a potent Ca^2+^-releasing secondary messenger that was firstly isolated from sea urchin eggs extracts. To obtain cpIPP the “northern” ribose of cADPR was replaced by a pentyl chain and the pyrophosphate moiety by a phophono-phosphate anhydride. The effect of the presence of the new phosphono-phosphate bridge on the intracellular Ca^2+^ release induced by cpIPP was assessed in PC12 neuronal cells in comparison with the effect of the pyrophosphate bridge of the structurally related cyclic N1-butylinosine diphosphate analogue (cbIDP), which was previously synthesized in our laboratories, and with that of the linear precursor of cpIPP, which, unexpectedly, revealed to be the only one provided with Ca^2+^ release properties.

## 1. Introduction

**Figure 1:**
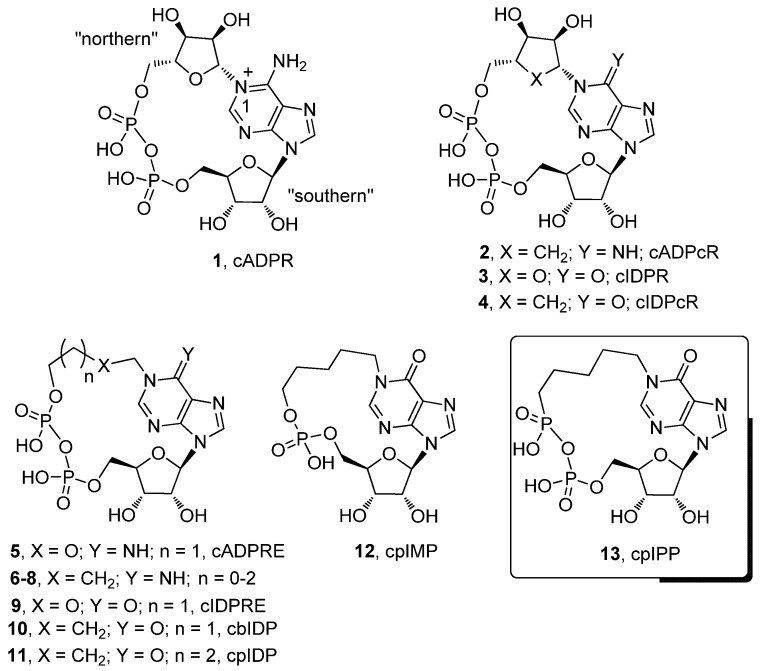
Some representative cyclic ADP-ribose (cADPR) analogues.

Cyclic ADP-ribose (cADPR, **1**, Figure 1) is a natural occurring metabolite of NAD^+^ that is capable of mobilizing Ca^2+^ ions from intracellular stores. cADPR was firstly isolated from sea urchin eggs extracts [1,2,3,4,5,6], but it was later established that it is also produced in many other mammalian cells, including pancreatic β-cells, T-lymphocytes, smooth and cardiac muscle cells, and cerebellar neurons, acting as a Ca^2+^-mobilizing agent [7,8,9].

For this activity, cADPR has been classified as a second messenger that, activating the ryanodine receptors of the sarcoplasmic reticulum, is able to mobilize the calcium ions from the intracellular stores [10]. cADPR is involved in many physiological processes that are related to the variation of the Ca^2+^ concentration, such as the synaptic homeostasis in neurons [11], as well as fertilization and cellular proliferation [12,13,14]. The conversion of the NAD^+^ into the 18-membered cyclic product (i.e., cADPR) has been attributed to the activity of the enzyme ADP-ribosyl cyclase, which was first purified from the marine mollusc *Aplysia Californica* [15]. In mammalian cells, the multifunctional transmembrane glycoprotein CD38 possesses this cyclase activity that converts the NAD^+^ into cADPR and catalyses the inverse hydrolytic reaction that produces adenosine 5’-diphosphate ribose (ADPR) [16,17,18].

In the last three decades, cADPR has been at the centre of numerous studies that were aimed at understanding its mechanism of action (the target proteins have not been yet completely identified) and at the production of potential drug candidates acting on calcium responsive physio-pathological processes. cADPR possesses a labile N1-glycosidic bond that is easily hydrolysed in aqueous solutions and in physiological conditions to the inactive ADPR [19]. To overcome this problem, most of the cADPR analogues so far prepared contain chemical modifications aimed at stabilizing the N1-ribosyl linkage and at exploring the structure activity relationship of this metabolite [20,21].

Many stable analogues of cADPR have been so far prepared by chemoenzymatic and chemical methods. The chemoenzymatic methods essentially follow a pathway that cyclizes a chemically modified NAD^+^ by the action of the *Aplysia Californica* ADP-ribosyl cyclase [15]. However, the specificity of the cyclase activity on the NAD^+^ analogues considerably limits the use of this strategy. On the contrary, the total chemical synthesis allows for the introduction of severe structural changes in the cADPR analogues [22].

The structural modifications so far proposed regard the northern and southern ribose, the purine base and the pyrophosphate moiety. Many of these cADPR analogues have also been biologically tested, thus allowing for the definition of some structure activity relationships (SAR) among the derivatives. For example, the substitution of the northern ribose with a carbocyclic ribose produces the stable mimic **2** (cADPcR) [23], which is more active than cADPR in the sea urchin egg homogenates and in neuronal cells, but results almost inactive in the T cell system [24]. Very recently, Shuto et al. reported on the synthesis and Ca^2+^ mobilizing activity of cyclic ADP-4-thioribose (cADPtR), in which the N1-ribose of cADPR is linked to 4-thioribose. cADPtR proved to be a biologically stable mimic of cADPR, as active as the latter in mobilizing Ca^2+^ ions in various cell systems [25].

In other cADPR analogues, the adenine base was replaced by hypoxanthine to get cIDPR (**3**) [26], or the corresponding N1-carbocyclic derivative cIDPcR (**4**) [27]. In Jurkat T cells, cIDPR showed almost the same Ca^2+^ mobilizing activity as cADPR. Conversely, cIDPcR resulted inactive in the same system up to 500 µM. 

In other studies, the northern ribose was replaced by more flexible moieties, such as the ethoxy-alkyl or alkyl chains, both for the adenine (**5**–**8**) and inosine series (**9**). Compound **5** (cyclic adenosine 5’-diphosphate-ribose-ether, cADPRE) and its N1-alkyl congeners (**6**–**8**) permeate the plasma membrane and are weak agonists of cADPR in human Jurkat T cells [28,29]. The inosine analogue **9** (cIDPRE) [30] possesses an activity on intact human Jurkat-T-lymphocytes releasing Ca^2+^ ions from intracellular stores.

Many cADPR analogues also contain modifications on the C-8 purine position, also in combination with modifications in the “northern” ribose. For example, atoms or groups like Cl, Br, NH_2_, N_3_, CF_3_, SPh, and OCH_3_ have been introduced on the C-8 purine position [31,32,33].

In the last years, we reported on the syntheses of several cIDPR analogues, focusing our attention on derivatives containing alkyl chains in place of the “northern” (**10**–**11**) or “southern” ribose [34,35]. Among these, the N1-pentyl derivative **11** showed an interesting Ca^2+^ releasing activity in PC12 cells that were differentiated in neurons with the use of nerve growth factor (NGF). Furthermore, we also found a significant Ca^2+^ releasing activity for the derivative **12**, in which the pyrophosphate moiety was replaced by a monophosphate function. In the present study, to further explore the role of the pyrophosphate group in the biological activity of cADPR analogues, we report on the synthesis and chemical and biological characterization of a new cyclic N1-pentyl inosine phosphono-phosphate anhydride analogue, cpIPP (**13**), in which the northern phosphate of **11** was replaced by a C-phosphonate moiety. This kind of modification is unprecedented and adds a further piece of information on the SAR of Ca^2+^ releasing cADPR analogues. Furthermore, the phosphono-phosphate anhydride moiety is a quite rare function in synthetic compounds, that, at the best of our knowledge, has never been explored in biologically active compounds. The Ca^2+^ mobilizing activities in PC12 cells of compounds **13** and of its linear precursor, as well as of the previously synthesized cyclic N1-butyl derivative **10** [35], which shares with the compound **13** the ring size, are also reported.

## 2. Results and Discussion

### 2.1. Chemistry

**Scheme 1:**
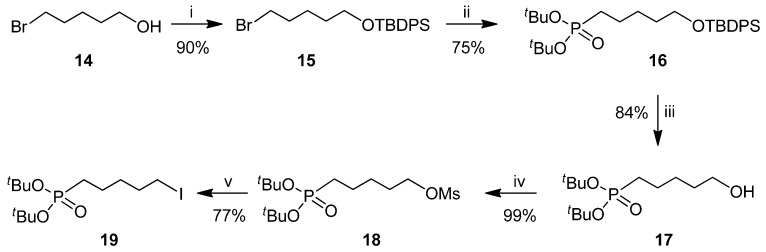
Reagents and conditions. (**i**) ref. [37]; (**ii**) NaH, HPO(O*^t^*Bu)_2_, DMF, 0 °C, 16 h; (**iii**) TBAF, THF, r.t., 2 h; (**iv**) mesyl chloride (MsCl), TEA, CH_2_Cl_2_, 0 °C, 1 h; (**v**) KI, DMF, 70 °C, 5 h.

The key step for the preparation of all cADPR/cIDPR analogues is the macrocyclization reaction via pyrophosphate bond formation, which is usually performed by joining the two phosphate moieties at the end of a multistep synthesis [23]. For the construction of the 18-membered ring in **13**, we have designed a convergent synthetic strategy that exploited the installation of a preformed phosphono-pentyl chain (**17** or **19**, Scheme 1) on the N1 position of inosine by a N-alkylation reaction. The synthesis of the phosphono-alkyl chain started from the commercially available 5-bromo-pentan-1-ol (**14**), which was silylated (**15**) [36] and then reacted with the sodium salt of di-*tert*-butyl phosphonate (generated in situ by reaction between NaH and di-*tert-*butyl phosphite), thus obtaining the intermediate **16** in very high yields. The deprotection of silyl ether in **16** with tetrabutylammonium fluoride (TBAF) allowed the quantitative recovery of the alcohol **17**. The latter was converted into the iodide **19**, passing through the mesylate (Ms) intermediate **18** in high yields. 

**Scheme 2:**
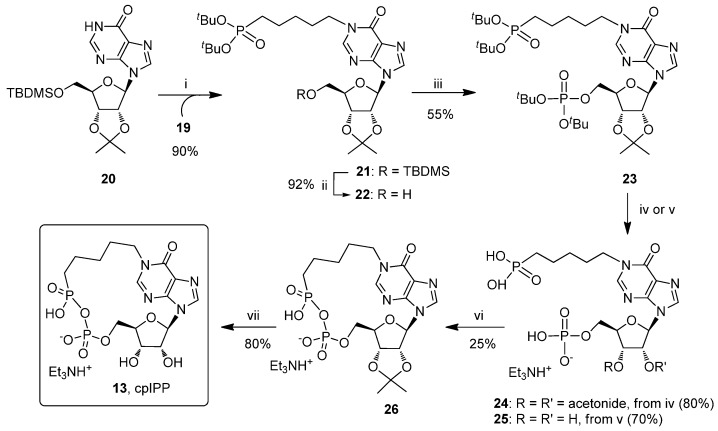
Reagents and conditions. (**i**) **19**, K_2_CO_3_, DMF, r.t., 16 h; (**ii**) TBAF, THF, r.t., 1 h; (**iii**) (**a**) (*^t^*BuO)_2_PN(*^i^*Pr)_2_, 1-*H*-tetrazole, THF, r.t., 4 h, (**b**) *^t^*BuOOH, THF, r.t., 1 h; (**iv**) 5% TFA in CH_2_Cl_2_, 0 °C, 5 h; (**v**) 50% TFA in H_2_O, r.t., 4 h; (**vi**) EDC, DMF, r.t., 72 h.; (**vii**) 60% HCO_2_H in H_2_O, r.t., 16 h.

As regarded the following N1-alkylation of the hypoxanthine base, it has to be considered that the purine is an ambident nucleophile that can lead to the formation of the N1 and O6 alkylated isomers, whose ratio depends on the electrophilicity of the carbon atom that undergoes the nucleophilic attack [27,37]. At first, the alkylation of the N1 purine position was performed under the Mitsunobu conditions, [38] by reacting the alcohol **17** with the nucleoside **20** (Scheme 2) [36] in the presence of triphenylphosphine (PPh_3_) and diethylazodicarboxylate (DEAD) under different experimental conditions (data not shown). However, the reaction yields were low for both isomers, with the preponderance of the O6-alkylated isomer (70–80% relative to the N1-alkyl isomer). The absence of N1 regioselectivity could be a consequence of the favoured reaction between the hard alkoxy-phosphonium cation intermediate and the nucleobase O6 hard atom [39]. The two isomers were clearly distinguished by comparison of their ^1^H-NMR spectra. In fact, in the O6 isomer the CH_2_ attached to the purine O6 atom resonated as a triplet at 4.5 ppm, whereas in the N1 isomer (**21**) the CH_2_ attached to the purine N1 atom resonated as a multiplet at 4.0 ppm, according to our previous findings [40,41,42,43,44,45]. With the aim of improving the yield and the regioselectivity of the reaction toward the required N1 alkylated isomer **21**, the reactivity of the soft iodide **19** was explored. At this stage, a careful selection of the alkaline conditions was crucial for the success of the reaction. Hyde, R. M. et al. [46] reported that 1,8-diazabicyclo[5.4.0]undec-7-ene (DBU) was very efficient to obtain N1-allyl inosine starting from allyl bromide and inosine, but in our case, compound **21** could be isolated from a complex reaction mixture only in a 14% yield. A similar result was also observed using the tertiary amines triethylamine (TEA) and *N*,*N*-diisopropylethylamine (DIPEA). Pleasingly, the use of K_2_CO_3_ pushed the reaction to completeness with the recovery of the N1-alkylated isomer **21** in an 90% yield. This reaction represents a mild and high yielding synthetic alternative for the inosine N1-alkylation.

Thereafter, the ribose *tert*-butyldimethylsilyl (TBDMS) group was quantitatively removed from the latter derivative with TBAF, thus obtaining the compound **22** that was phosphorylated in 55% yield by reaction with di-*tert*-butyl *N*,*N*-diisopropylphosphoramidite [(*^t^*BuO)_2_PN(*^i^*Pr)_2_] and 1-*H*-tetrazole, followed by *tert*-butyl hydroperoxide (*^t^*BuOOH) oxidation, to give the derivative **23**. Next, the protecting groups of the phosphate and phosphonate moieties in the product **23** were removed by reaction with trifluoroacetic acid (TFA) in anhydrous dichloromethane (DCM), yielding compound **24** (80% yield). This treatment preserved the ribose acetonide protecting group, which was strictly necessary for the success of the final macrocyclization reaction. Conversely, the treatment of compound **23** with aqueous TFA solution afforded the fully deprotected nucleotide **25**. The compound **24**, dissolved in DMF at the final concentration of 2 mM was treated with 1-ethyl-3-(3-dimethylaminopropyl)carbodiimide (EDC) (1.2 equiv.) and the reaction allowed to stand at room temperature for 72 h. From this mixture, it was possible to isolate the cyclic compound **26** in a 25% yield. The diasterotopicity of CH_2_N1 protons in the ^1^H-NMR spectrum (resonating as two doublets of doublets of doublets at 4.45 and 4.05 ppm) and the presence of two doublets in the ^1^H-decoupled ^31^P-NMR spectrum (19.3 ppm for the phosphonate and −11.2 ppm for the phosphate) of the compound **26** were clear evidences of the 18-membered ring formation. On the contrary, for the linear precursor **24**, the CH_2_N1 protons appeared as a triplet at 4.06 ppm in the ^1^H-NMR spectrum, whereas the phosphorous atoms of the phosphonate and phosphate groups resonated as two singlets at 28.2 and 1.0 ppm, respectively, in the ^1^H-decoupled ^31^P-NMR spectrum. Finally, the treatment of compound **26** with aqueous 60% HCO_2_H afforded the cpIPP **13** in 80% yield (7.3% overall yield starting from compound **20**). In the analogue **13**, the N1–C bond and the phosphono-phosphate anhydride moiety revealed to be stable to the hydrolysis, as evidenced by HPLC analyses, which indicated the absence of any degradation product after its dissolution in water at pH = 7 (data not shown).

### 2.2. Biology

**Figure 2:**
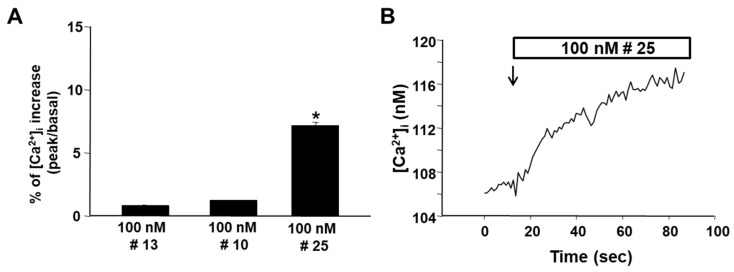
Effect of compounds **10**, **13** and **25** on [Ca^2+^]_i_ in NGF-differentiated PC12 cells. Panel (**A**): quantification of [Ca^2+^]_i_ increase calculated as the percentage change of plateau/basal value after the addition of compounds **10**, **13** and **25**. Each bar represents the mean (±S.E.M.) of the values obtained in three independent experimental sessions. For each experiment, 10 to 40 individual cells were monitored. *, *p* < 0.05 versus basal level. Panel (**B**): representative single-cell trace of the effect of compound **25** (100 nM,) on [Ca^2+^]_i_.

Exogenous administration of cADPR did not produce changes in [Ca^2+^]_i_ for the low ability of the metabolite to cross the membranes. To overcome this problem, the three more lipophilic derivatives **10**, **13**, and **25** were synthesized and tested. These analogues were perfused at the concentration of 100 nM in a Krebs-Ringer saline solution to PC12 cells previously differentiated in neurons with NGF. While the cyclic compounds **10** and **13** failed to modify [Ca^2+^]_i_, the linear precursor **25** caused a fast but low increase in [Ca^2+^]_i_ (Figure 2).

However, this effect was lower than that previously obtained with cyclic analogues **11** and **12 [36]**, likely because of a reduced ability to cross the membranes or to an inefficacious interaction with the intracellular receptor.

### 2.3. Conformational Analyses

Prompted by the results of biological studies, we asked whether the differences in activity that was shown by compounds **10** and **13** with respect to the analogue **11** were due to a different conformational behaviour. We therefore sampled the conformational space of compounds **10**, **11,** and **13** by using the macrocycle conformational sampling routine implemented in Maestro 11.2 (Schroedinger Inc.) [47]. Conformational ensembles that were obtained from the macrocycle search evidenced that all the compounds covered the same conformational space (Figure S1, Supplementary Information), albeit the phosphonate/phosphate derivative (**13**) clearly showed a lower number of low energy conformers. Interestingly, ring dimension does not seem to play a key role, since both pyrophosphate derivatives (**10** and **11**) showed a similar number of conformers.

**Figure 3:**
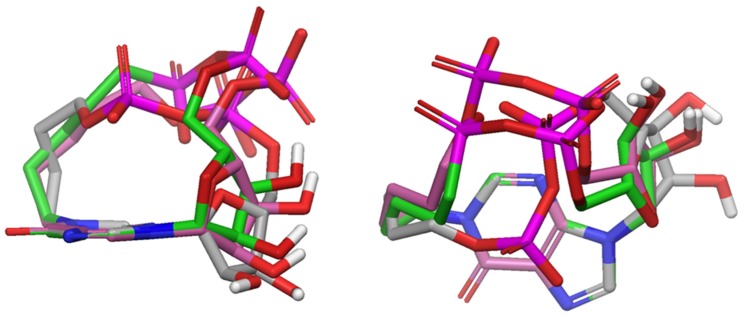
Side (**left**) and top (**right**) view of the lowest energy conformers of cbIDP (**10**, grey), cpIDP (**11**, pink) and cpIPP (**13**, green) superimposed on the hypoxanthine ring. Non-polar hydrogens were omitted for sake of clarity. Oxygens were reported in red, nitrogens in blue, phosphates in magenta, hydrogens in white.

**Figure 4:**
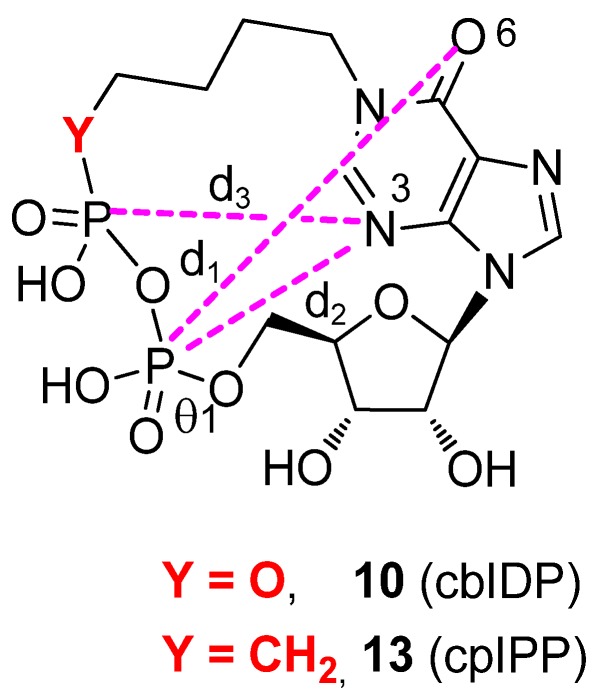
Definition of the geometrical descriptors. d_1_: distance between O6 purine atom and P atom of the southern phosphate; d_2_: distance between N3 purine atom and P atom of the southern phosphate; d_3_: distance between N3 purine atom and P atom of the northern phosphate/phosphonate.

The comparison of the lowest energy conformers of the compounds **10**, **11,** and **13** (Figure 3) showed a different displacement of the pyrophosphate (**10**, **11**) or phosphonate/phosphate (**13**) groups with respect to the hypoxanthine ring, leading to different structural features that could play a key role in the biological activity. In particular, the southern phosphate group of **11** is generally farther from the centre of the ring when compared to compound **10** and **13**, as shown by the analysis of distances d_1_, d_2_, and d_3_ (describing the relative position of the phosphate/phosphonate groups with respect to the hypoxanthine ring defined in Figure 4; Tables S1 and S2, Supporting Information). Nevertheless, in our opinion, the differences that were observed in conformational behaviour of compounds **11** and **13** were not so relevant to be a discriminant for the biological activity. On the other hand, the calculation of ALogP (Table S1, Supplementary Information) showed lower lipophilicity for the compounds **10** and **13**. On the basis of these considerations, we hypothesise that the lack of activity characterizing compounds **10** and **13** may be due to a combination of factors, including conformational flexibility that could affect interaction with the intracellular target and a reduced ability to cross membranes.

## 3. Experimental Section

### 3.1. General Experimental Procedures

All the reagents and solvents for the chemical syntheses were obtained from commercial sources and were used without further purification. ^1^H- and ^13^C-NMR experiments were performed using Varian Inc. NMR spectrometers (Mercury Plus 400 MHz, ^UNITY^INOVA 500 MHz and ^UNITY^INOVA 700 MHz, Varian Inc., Lake Forest, CA, USA) and CDCl_3_, CD_3_OD and D_2_O as solvents. NMR chemical shifts are reported in parts per million (*δ*) relative to residual solvents signals: CHCl_3_ 7.27, CD_2_HOD 3.31, HOD 4.80 for ^1^H-NMR, and CDCl_3_ 77.0, CD_3_OD 49.0 for ^13^C NMR. The ^1^H-decoupled ^31^P-NMR experiments were carried out on the ^UNITY^INOVA 500 MHz instrument in CDCl_3_ and D_2_O solvents using 85% H_3_PO_4_ as external standard (0 ppm).

The ^1^H-NMR chemical shifts were assigned through 2D NMR experiments. High performance liquid chromatography (HPLC) was performed using a Jasco UP-2075 Plus (Jasco Europe s.r.l., Cremella (LC), Italy) pump equipped with a Jasco UV-2075 Plus UV detector (Jasco Europe s.r.l., Cremella (LC), Italy) and a Merck Purosphere Star (Merck & Co., Kenilworth, NJ, USA) 4.8 × 150 mm C-18 reversed-phase column (particle size 5 µm) eluted with a linear gradient of CH_3_CN in 0.1 M triethylammonium bicarbonate (TEAB) buffer (from 0 to 100% in 45 min, flow 1.3 mL/min). UV spectra were recorded on a Jasco V-530 UV spectrophotometer (Jasco Europe s.r.l., Cremella (LC), Italy). ESI-MS spectra were recorded on an AB SCIEX QTRAP 4000 LC-MS/MS system. Column chromatography was carried out on silica gel-60 (Merck, 0.063-0.200 mm). Analytical TLC analyses were performed using F254 silica gel plates (0.2 mm thick, Merck). TLC spots were detected under UV light (254 nm).

### 3.2. Synthesis and Charcterization of Compounds ***13***, ***16***, ***17***, ***19*** and ***21***–***26***

Compound **16**: To an ice-cooled solution of di-*tert*-butyl phosphite (0.8 mL, 1.3 mmol) in dry DMF (3 mL) under nitrogen atmosphere, NaH (96 mg, 4.0 mmol) was added. After 10 min, compound **15** (0.81 g, 2.0 mmol) dissolved in dry DMF (3 mL) was added *via* cannula. The reaction mixture was warmed to room temperature and stirred for 16 h (TLC monitoring: *n*-hexane/AcOEt, 8:2), quenched with CH_3_OH, diluted with AcOEt (50 mL), and washed with brine (50 mL). The organic layer was dried over anhydrous Na_2_SO_4_, filtered, and evaporated under reduced pressure. The residue was purified over a silica gel column eluted with increasing amounts of AcOEt in *n*-hexane (up to 70%) to afford pure **16**. Oil (0.78 g, 75% yield). ^1^H NMR (500 MHz, CDCl_3_) δ 7.81–7.57 (m, 4H, arom.), 7.38 (dt, *J* = 13.7, 6.8 Hz, 6H, arom.), 3.65 (t, *J* = 6.4 Hz, 2H, CH_2_O), 1.70–1.36 (complex signal, 26H, 4 × CH_2_ and 2 × O*^t^*Bu), 1.04 (s, 9H, *^t^*Bu). ^13^C NMR (100 MHz, CDCl_3_) δ 135.4, 133.9, 129.4, 127.4, 81.1, 81.0, 63.6, 32.1, 30.3, 30.2 (d, *J* = 145.2 Hz), 30.2 (2C), 26.8 (d, *J* = 16.8 Hz), 23.2 (d, *J* = 6.0 Hz). ^31^P NMR (202 MHz, CDCl_3_) δ 25.2 (s). ESI-MS *m/z* 519 ([M + H]^+^, C_29_H_48_O_4_PSi, requires 519).

Compound **17**: To a solution of compound **16** (0.50 g, 0.96 mmol) in dry THF (5 mL), TBAF (1.1 mL, 1.1 mmol) was added dropwise. The reaction mixture was stirred at room temperature for 2 h (TLC monitoring: AcOEt) and then evaporated under reduced pressure. The residue was purified over a silica gel column eluted with increasing amounts of CH_3_OH in AcOEt (up to 1%) to afford pure **17**. Oil (0.22 g, 84% yield). ^1^H NMR (400 MHz, CDCl_3_) δ 3.61 (t, *J* = 6.4 Hz, 2H, CH_2_O), 2.58 (bs, 1H, OH), 1.70–1.34 (complex signal, 26H, 4 × CH_2_ and 2 × O*^t^*Bu). ^13^C NMR (100 MHz, CDCl_3_) δ 81.4, 81.3, 62.4, 32.2, 30.4, 30.3, 30.1 (d, *J* = 145.1 Hz), 26.6 (d, *J* = 17.8 Hz), 23.1 (d, *J* = 6.2 Hz). ^31^P NMR (202 MHz, CDCl_3_) δ 25.1 (s). ESI-MS *m/z* 303 ([M + Na]^+^, C_13_H_29_NaO_4_P, requires 303).

Compound **19**: To an iced-cooled solution of compound **17** (0.22 g, 0.80 mmol) in dry CH_2_Cl_2_ (4 mL), TEA (0.075 mL, 0.96 mmol), and then MsCl (0.22 mL, 1.6 mmol) were added. The reaction mixture was stirred at 0 °C for 1 h (TLC monitoring: AcOEt/CH_3_OH, 98:2), quenched with H_2_O, diluted with CH_2_Cl_2_ (30 mL) and washed with brine (30 mL). The organic layer was dried over anhydrous Na_2_SO_4_, filtered and evaporated under reduced pressure. The residue was dissolved in dry DMF (8 mL) and KI (0.40 g, 2.4 mmol) was added. The reaction mixture was stirred at 70 °C for 5 h (TLC monitoring: *n*-hexane/AcOEt, 1:1) and then cooled to room temperature. Water (30 mL) was added and the product was extracted with Et_2_O (3 × 30 mL). The combined organic extracts were washed with water brine, dried over anhydrous Na_2_SO_4_, filtered and concentrated under reduced pressure to afford a crude that was purified by silica gel column eluted with increasing amounts of AcOEt in *n*-hexane (up to 50%) to afford pure **19**. Oil (0.24 g, 77% yield over two steps). ^1^H NMR (400 MHz, CDCl_3_) δ 3.16 (t, *J* = 7.0 Hz, 2H, CH_2_I), 1.86–1.76 (m, 2H, CH_2_), 1.67–1.35 (complex signal, 24H, 3 × CH_2_ and 2 × O*^t^*Bu). ^13^C NMR (100 MHz, CDCl_3_) δ 81.3, 81.2, 62.4, 33.1, 31.3 (d, *J* = 16.7 Hz), 30.4, 30.3, 30.0 (d, *J* = 145.4 Hz), 22.4 (d, *J* = 6.0 Hz), 6.5. ^31^P NMR (202 MHz, CDCl_3_) δ 24.6 (s). ESI-MS *m/z* 413 ([M + Na]^+^, C_13_H_28_INaO_3_P, requires 413).

Compound **21**: To a solution of compound **20** (0.12 g, 0.28 mmol) in dry DMF (1 mL), anhydrous K_2_CO_3_ (0.077 g, 0.56 mmol) was added. After 10 min, compound **19** (0.22 g, 0.61 mmol), dissolved in dry DMF (1 mL), was added. The reaction mixture was stirred at room temperature for 16 h (TLC monitoring: CHCl_3_/CH_3_OH, 95:5), diluted with AcOEt (20 mL) and washed with brine (20 mL). The organic layer was dried over anhydrous Na_2_SO_4_, filtered, and evaporated under reduced pressure. The residue was purified over a silica gel column eluted with increasing amounts of CH_3_OH in CHCl_3_ (up to 1%) to afford pure **21**. Oil (0.17 g, 90% yield). ^1^H NMR (400 MHz, CDCl_3_) δ 7.96 (s, 1H, 8-H), 7.91 (s, 1H, 2-H), 6.04 (bs, 1H, 1′-H), 5.05-4.99 (s, 1H, 2′-H), 4.86-4.80 (s, 1H, 3′-H), 4.34 (bs, 1H, 4′-H), 4.07-3.88 (m, 2H, CH_2_N), 3.79 (dd, *J* = 3.4, 11.5 Hz, 1H, 5′-H_a_), 3.75 (dd, *J* = 3.7, 11.5 Hz, 1H, 5′-H_b_), 1.78–1.65 (m, 2H, CH_2_), 1.60–1.46 (complex signal, 7H, 2 × CH_2_ and CH_3_), 1.42–1.36 (complex signal, 20H, CH_2_ and 2 × O*^t^*Bu), 1.32 (s, 3H, CH_3_), 0.77 (s, 9H, Si*^t^*Bu), −0.04 (s, 3H, CH_3_), −0.05 (s, 3H, CH_3_). ^13^C NMR (100 MHz, CDCl_3_) δ 156.3, 147.1, 146.8, 138.2, 124.8, 114.1, 91.2, 87.0, 85.3, 81.3, 81.2, 81.1, 63.4, 46.7, 30.4, 30.3, 30.0 (d, *J* = 145.6 Hz), 29.4, 27.3 (d, *J* = 17.0 Hz), 27.2, 25.8, 25.3, 23.1 (d, *J* = 5.9 Hz), −5.4, −5.6. ^31^P NMR (202 MHz, CDCl_3_) δ 24.4 (s). ESI-MS *m/z* 707 ([M + Na]^+^, C_32_H_57_N_4_NaO_8_PSi, requires 707). 

Compound **22**: To a solution of compound **21** (0.15 g, 0.22 mmol) in dry THF (5 mL), TBAF (0.3 mL, 0.26 mmol) was added dropwise. The reaction mixture was stirred at room temperature for 1 h (TLC monitoring: CHCl_3_/CH_3_OH, 95:5) and then evaporated under reduced pressure [48,49]. The residue was purified over a silica gel column eluted with increasing amounts of CH_3_OH in CHCl_3_ (up to 2%) to afford pure **22**. Oil (0.13 g, 92% yield). ^1^H NMR (400 MHz, CDCl_3_) δ 8.11 (bs, 1H, 8-H), 8.01 (s, 1H, 2-H), 5.92 (d, *J* = 3.9 Hz, 1H, 1′-H), 5.15–5.08 (m, 1H, 2′-H), 5.07-5.02 (m, 1H, 3′-H), 4.52 (bs, 1H, 4′-H), 4.11–4.00 (m, 2H, CH_2_N), 3.95 (d, *J* = 12.3 Hz, 1H, 5′-H_a_), 3.79 (d, *J* = 12.4 Hz, 1H, 5′-H_b_), 3.19 (bs, 1H, OH), 1.85–1.73 (m, 2H, CH_2_), 1.65–1.55 (complex signal, 7H, 2 × CH_2_ and CH_3_), 1.51–1.40 (complex signal, 20H, CH_2_ and 2 × O*^t^*Bu), 1.37 (s, 3H, CH_3_). ^13^C NMR (100 MHz, CDCl_3_) δ 155.7, 147.5, 146.1, 139.7, 125.2, 114.2, 93.8, 86.5, 84.1, 81.6, 81.4, 63.0, 47.1, 30.4, 30.0 (d, *J* = 146.0 Hz), 29.4, 27.4, 27.3 (d, *J* = 16.3 Hz), 25.8, 25.2, 23.1 (d, *J* = 5.5 Hz). ^31^P NMR (202 MHz, CDCl_3_) δ 24.4 (s). ESI-MS *m/z* 593 ([M + Na]^+^, C_26_H_43_N_4_NaO_8_P, requires 593). 

Compound **23**: To a solution of compound **22** (0.11 g, 0.19 mmol), in dry THF (5 mL), 1-*H*-tetrazole (0.094 g, 1.3 mmol), and then *^i^*Pr_2_NP(*^t^*BuO)_2_ (0.84 mL, 2.6 mmol) were added under nitrogen atmosphere [50,51]. The reaction mixture was stirred at room temperature for 4 h (TLC monitoring: CHCl_3_/CH_3_OH, 95:5) and then *^t^*BuOOH (0.35 mL of a solution 5.5 M in decane, 1.9 mmol) was added at room temperature. After 1h (TLC monitoring: CHCl_3_/CH_3_OH, 95:5) the reaction mixture was evaporated under reduced pressure, diluted with AcOEt (30 mL), and washed with brine (30 mL). The organic layer was dried over anhydrous Na_2_SO_4_, filtered and evaporated under reduced pressure. The residue was purified over a silica gel column eluted with increasing amounts of CH_3_OH in CHCl_3_ (up to 5%) to afford pure **23**. Oil (76 mg, 55% over two steps). ^1^H NMR (400 MHz, CDCl_3_) δ 8.01 (bs, 1H, 8-H), 7.98 (s, 1H, 2-H), 6.10 (bs, 1H, 1′-H), 5.22–5.17 (m, 1H, 2′-H), 5.04–4.99 (m, 1H, 3′-H), 4.47 (bs, 1H, 4′-H), 4.18–4.11 (m, 2H, CH_2_N), 4.09–3.99 (m, 2H, 5′-H_a,b_), 1.83–1.73 (m, 2H, CH_2_), 1.64–1.58 (complex signal, 7H, 2 × CH_2_ and CH_3_), 1.49–1.45 (complex signal, 29H, CH_2_ and 3 × O*^t^*Bu), 1.42 (s, 9H, O*^t^*Bu), 1.37 (s, 3H, CH_3_). ^13^C NMR (100 MHz, CDCl_3_) δ 156.3, 147.3, 146.9, 138.6, 124.5, 114.6, 90.7, 84.9, 84.6, 83.1, 83.0, 81.5, 81.4, 81.3, 66.0, 46.9, 30.4, 30.0 (d, *J* = 145.4 Hz), 29.8, 29.5, 27.4 (d, *J* = 17.1 Hz), 27.2, 25.3, 23.1 (d, *J* = 5.5 Hz). ^31^P NMR (202 MHz, CDCl_3_) δ 24.4 (s), –8.7 (s). ESI-MS *m/z* 785 ([M + Na]^+^, C_34_H_60_N_4_NaO_11_P_2_, requires 785). 

Compound **24**: To an ice-cooled solution of compound **23** (50 mg, 0.065 mmol) in dry CH_2_Cl_2_ (0.95 mL) TFA (0.050 mL) was added. The reaction mixture was stirred at 0 °C for 5 h (TLC monitoring: *^i^*PrOH/NH_3_(aq)/H_2_O, 6:3:1), warmed to room temperature and evaporated under reduced pressure. The residue was dissolved in TEAB 0.1 M buffer, passed over a polyvinylidene difluoride (PVDF) 0.45 μm filter and purified by HPLC (see General experimental procedures). The fractions containing the triethylammonium salt of compound **24** (*t_R_* = 21.3 min) were collected and lyophilized. Amorphous white solid (33 mg, 80% yield). ^1^H NMR (400 MHz, D_2_O) δ 8.40 (bs, 1H, 8-H), 8.39 (s, 1H, 2-H), 6.30 (d, *J* = 3.1 Hz, 1H, 1′-H), 5.43 (dd, *J* = 6.1, 3.1 Hz, 1H, 2′-H), 5.21 (dd, *J* = 6.1, 2.2 Hz, 1H, 3′-H), 4.70-4.65 (m, 1H, 4′-H), 4.21-4.10 (m, 2H, 5′-H_a,b_), 4.06 (t, *J* = 4.5 Hz, 2H, CH_2_N), 3.21 (q, *J* = 7.3 Hz, 6H, 3 × CH_2_, triethylammonium), 1.88–1.78 (m, 2H, CH_2_), 1.67 (s, 3H, CH_3_), 1.65–1.54 (complex signal, 4H, 2 × CH_2_), 1.50–1.41 (complex signal, 5H, CH_2_ and CH_3_), 1.29 (t, *J* = 7.3 Hz, 9H, 3 × CH_3_, triethylammonium). ^13^C NMR (175 MHz, D_2_O) δ 158.1, 148.8, 147.5, 140.1, 123.5, 114.8, 90.5, 85.2 (d, *J* = 8.8 Hz), 84.1, 81.4, 64.7 (*J* = 4.1 Hz), 47.2, 46.6, 28.2, 27.6 (d, *J* = 144.3), 27.0 (d, *J* = 17.5 Hz), 26.0, 24.1, 22.6 (d, *J* = 4.2 Hz), 8.1. ^31^P NMR (202 MHz, D_2_O) δ 28.2 (s), 1.0 (s). ESI-MS *m/z* 537 ([M − H]^−^, C_18_H_27_N_4_O_11_P_2_, requires 537).

Compound **25**: To a solution of compound **23** (10 mg, 0.013 mmol) in H_2_O (0.20 mL) TFA (0.20 mL) was added. The reaction mixture was stirred at room temperature for 4 h (TLC monitoring: *^i^*PrOH/NH_3_(aq)/H_2_O, 6:3:1) and then evaporated under reduced pressure. The residue was dissolved in TEAB 0.1 M buffer, passed over a PVDF 0.45 μm filter and then purified by HPLC (see General experimental procedures). The fractions containing the triethylammonium salt of compound **25** (*t_R_* = 19.1 min) were collected and lyophilized. Amorphous white solid (4.5 mg, 70% yield). ^1^H NMR (500 MHz, D_2_O) δ 8.47 (bs, 1H, 8-H), 8.42 (s, 1H, 2-H), 6.15 (d, *J* = 5.6 Hz, 1H, 1′-H), 4.80 (1H, 2′-H, covered by residual solvent signal), 4.53–4.50 (m, 1H, 3′-H), 4.42–4.38 (m 1H, 4′-H), 4.20–4.10 (complex signal, 4H, 5′-H_a,b_ and CH_2_N), 3.22 (q, *J* = 7.3 Hz, 6H, 3 × CH_2_, triethylammonium), 1.87–1.79 (m, 2H, CH_2_), 1.66-1.54 (complex signal, 4H, 2 × CH_2_), 1.50–1.42 (m, 2H, CH_2_), 1.30 (t, *J* = 7.3 Hz, 9H, 3 × CH_3_, triethylammonium) ^13^C NMR (100 MHz, D_2_O) δ 158.1, 148.9, 147.9, 139.8, 123.4, 87.2, 84.1 (d, *J* = 8.5 Hz), 74.4, 70.3, 64.2 (d, *J* = 3.7 Hz), 47.3, 46.6, 28.2. 27.6 (d, *J* = 143.7 Hz), 27.0 (d, *J* = 17.1 Hz) 22.6 (d, *J* = 3.3 Hz), 8.1. ^31^P NMR (202 MHz, D_2_O) δ 27.4 (s), 1.7 (s). ESI-MS *m/z* 497 ([M − H]^−^, C_15_H_23_N_4_O_11_P_2_, requires 497).

Compound **26**: To a solution of compound **24** (20 mg, 0.031 mmol) in dry DMF (15 mL) EDC (0.012 g, 0.062 mmol) was added. The reaction mixture was stirred at room temperature for 72 h and evaporated under reduced pressure. The residue was dissolved in TEAB 0.1 M buffer, passed over a PVDF 0.45 μm filter and purified by HPLC (see General experimental procedures). The fractions containing the triethylammonium salt of compound **26** (*t_R_* = 23.8 min) were collected and lyophilized. Amorphous white solid (4.8 mg, 25% yield). ^1^H NMR (500 MHz, D_2_O) δ 8.49 (s, 1H, 8-H), 8.25 (s, 1H, 2-H), 6.37 (bs, 1H, 1′-H), 5.84 (d, *J* = 6.0 Hz, 1H, 2′-H), 5.40 (dd, *J* = 6.0, 2.2 Hz, 1H, 3′-H), 4.65–4.60 (m, 1H, 4′-H), 4.45 (ddd, *J* = 13.4, 5.6, 3.9 Hz, 1H, CH_a_N), 4.05 (ddd, *J* = 13.2, 8.9, 3.6 Hz, 1H, CH_b_N), 3.98–3.87 (m, 2H, 5′-H_a,b_), 3.22 (q, *J* = 7.3 Hz, 6H, 3 × CH_2_, triethylammonium), 2.00–1.86 (m, 2H, CH_2_), 1.66 (s, 3H, CH_3_), 1.61–1.51 (m, 2H, CH_2_), 1.50 (s, 3H, CH_3_), 1.45-1.32 (m, 4H, 2 × CH_2_), 1.30 (t, *J* = 7.3 Hz, 9H, 3 × CH_3_, triethylammonium). ^13^C NMR (175 MHz, D_2_O) δ 158.3, 148.4, 147.2, 141.7, 124.0, 114.0, 91.1, 87.2 (d, *J* = 10 Hz), 83.6, 81.5, 65.2 (d, *J* = 5.0 Hz), 48.0, 46.5, 28.2 (d, *J* = 140.1 Hz), 27.2 (d, *J* = 17.5 Hz), 26.3, 25.6, 22.7, 8.1. ^31^P NMR (202 MHz, D_2_O) δ 19.3 (d, ^2^*J*_P,P_ = 27.8 Hz), –11.4 (d, ^2^*J*_P,P_ = 27.2 Hz). ESI-MS *m/z* 519 ([M − H]^−^, C_18_H_25_N_4_O_10_P_2_, requires 519).

Compound **13** (cpIPP): A solution of compound **16** (3.0 mg, 0.0053 mmol) in aqueous 60% HCO_2_H (0.5 mL) was stirred at room temperature for 16 h and then evaporated under reduced pressure. The crude was dissolved in TEAB 0.1 M buffer, passed over a PVDF 0.45 μm filter and purified by HPLC (see General experimental procedures). The fractions containing the triethylammonium salt of compound **13** (*t_R_* = 19.8 min) were collected and lyophilized. Amorphous white solid (2.0 mg, 80% yield). ^1^H NMR (700 MHz, D_2_O) δ 8.47 (s, 1H, 8-H), 8.21 (s, 1H, 2-H), 6.06 (d, *J* = 3.5 Hz, 1H, 1′-H), 5.36 (dd, *J* = 3.6, 5.0 Hz, 1H, 2′-H), 4.80 (1H, 3′-H, covered by residual solvent signal), 4.34–4.28 (complex signal, 2H, 4′-H and CH_a_N), 4.18–4.13 (m, 1H, CH_b_N), 4.12–4.08 (m, 1H, 5′-H_a_), 4.07–4.03 (m, 1H, 5′-H_b_), 3.20 (q, *J* = 7.3 Hz, 6H, 3 × CH_2_, triethylammonium),1.95–1.86 (m, 2H, C*H*_2_CH_2_N), 1.60–1.52 (m, 1H, C*H*_a_CH_2_CH_2_N), 1.42–1.30 (m, 2H, PCH_2_), 1.29–1.22 (complex signal, 10H, 3 × CH_3_ triethylammonium and C*H*_b_CH_2_CH_2_N), 1.16–1.06 (m, 2H, PCH_2_C*H*_2_). ^13^C NMR (175 MHz, D_2_O) δ 158.2, 148.2, 147.6, 141.5, 123.9, 89.6, 83.4, 72.1, 69.6, 64.0, 47.6, 46.5, 27.8, 26.9, 25.9, 22.2, 8.1. ^31^P NMR (202 MHz, D_2_O) δ 19.3 (d, ^2^*J*_P,P_ = 26.4 Hz), −11.2 (d, ^2^*J*_P,P_ = 26.8 Hz). ESI-MS *m/z* 479 ([M − H]^−^, C_15_H_21_N_4_O_10_P_2_, requires 479). 

### 3.3. Cell Cultures and Intracellular [Ca^2+^]_i_ Measurement for Compounds ***10***, ***13*** and ***25***

PC12 cells, which grown on plastic dishes in RPMI medium (composed of 10% horse serum, 5% FBS, 100 UI/mL penicillin, and 100 μg/mL streptomycin) were differentiated in neurons with NGF (50 ng/mL; 7 days). Cells were cultured in the atmosphere of 5% CO_2_. Culture medium was changed every 2 days. Intracellular Ca^2+^ concentration ([Ca^2+^]_i_) was measured by single cell computer-assisted video-imaging [52]. Briefly, differentiated PC12 cells cultured on glass coverslips coated with poly-L-lysine (5 μg/mL) (Sigma, St. Louis, MO, USA) were loaded with 10 µM Fura-2AM for 1 h at 22 °C in Krebs-Ringer saline solution containing the following: 5.5 mM KCl, 160 mM NaCl, 1.2 mM MgCl_2_, 1.5 mM CaCl_2_, 10 mM glucose, and 10 mM HEPES-NaOH, pH 7.4. At the end of the loading period, the coverslips were placed in a perfusion chamber (Medical System, Greenvale, NY, USA), mounted on a Zeiss Axiovert 200 microscope (Carl Zeiss, Oberkochen, Germany) equipped with a FLUAR 40× oil objective lens. The experiments employed a digital imaging system that was composed of a MicroMax 512BFT cooled CCD camera (Princeton Instruments, Trenton, NJ, USA), LAMBDA 10-2 filter wheeler (Sutter Instruments, Novato, CA, USA), and Meta-Morph/MetaFluor Imaging System software (Universal Imaging, West Chester, PA, USA). After loading, cells were illuminated alternately at 340 and 380 nm by a Xenon lamp. The emitted light was passed through a 512 nm barrier filter. Fura-2AM fluorescence intensity was measured every 3s. Forty to sixty-five individual cells were selected and monitored simultaneously from each cover slip. Results are presented as the cytosolic Ca^2+^ concentration. Calibrations used the relation of Grynkiewicz et al. [53] assuming that the KD for Fura-2AM was 224 nM. 

### 3.4. Conformational Search

Calculations were performed using the Macromodel Conformational sampling from Schrödinger [47] using the OPLS 2005 force field and a generalized Born continuum solvent model (electrostatic treatment of water solvent). 10000 Large Scale Low Mode (LLMOD) steps were performed and normal modes were recalculated every time a new conformation was generated. Conformers with RMSD ≤ 0.75 Å were deemed redundant and removed. Only conformations within 10 kcal of the observed global minimum were retained.

## 4. Conclusions

cADPR is a ubiquitous second messenger responsible for Ca^2+^ mobilization from intracellular stores. Its instability in neutral aqueous conditions and its inability to cross the blood-brain barrier pushed chemists to develop synthetic or chemo-enzymatic strategies to obtain more stable and less polar analogues to better understand its physiological roles. In this frame, we have previously synthesized the cADPR analogue **11**, in which the “northern” ribose is replaced by a pentyl chain and the corresponding structurally reduced derivative **12** having a phosphodiester moiety in the place of the pyrophosphate one. When considering the encouraging biological results that were obtained by analogues **11** and **12** on PC12 neuronal cells and to better explore the exact role of the pyrophosphate in the Ca^2+^ releasing activity, herein we have reported on the synthesis of a more lipophilic and not-hydrolysable cADPR analogue **13** (cpIPP), containing the not so far explored phosphono-phosphate anhydride moiety. In accordance with our recent results, the final cyclization step was performed at the end of the synthesis by joining the fully deprotected C-phosphonate and phosphate functionalities. This reaction, even if low yielding, allowed for a rapid recovery of the cADPR derivative **13** for the evaluation of its biological activity. Preliminary experiments on PC12 neuronal cells have shown that the cyclic compounds **10** and **13** failed to modify the [Ca^2+^]_i_, whereas the linear compound **25** caused a fast but low increase in [Ca^2+^]_i_ if compared to the efficacy of the previously described cyclic analogues **11** and **12**. 

From these results, we could conclude that the pyrophosphate and the pentyl chain that is installed on the inosine N1 position are key elements for the Ca^2+^ releasing activity in PC12 neuronal cells of the cIDPR analogues having alkyl chains in the place of the “northern” ribose. Furthermore, the activity shown by the linear analogue **25**, in which the phosphonate and the phosphate moieties are not joined, paves the way for new studies that are aimed at the identification of the minimal structural unit required for the biological activity of linear analogues of IDPR.

## Supplementary Materials

The following are available online at http://www.mdpi.com/1660-3397/16/3/89/s1, S3–S12: copies of ^1^H-NMR and ^31^P-NMR spectra of compounds **13**, **16**, **17**, **19** and **21**–**26**; S13–S21: copies of ^13^C-NMR spectra of compounds **16**, **17**, **19** and **21**–**26**; S22: Table S1; S22: Figure S1; S23: Table S2.
