# Supplementary material for: Synthesis and Biological Evaluation of a New Structural Simplified Analogue of cADPR, a Calcium-Mobilizing Secondary Messenger Firstly Isolated from Sea Urchin Eggs
Source: Mar Drugs. 2018 Mar 10;16(3):89. doi: 10.3390/md16030089 (PMC5867633; doi:10.3390/md16030089)
Supplement: Supplementary file 1 [file marinedrugs-16-00089-s001.pdf]

Synthesis and biological evaluation of a new structural simplified analogue of cADPR, a calcium-mobilizing secondary messenger firstly isolated from sea urchin eggs

Stefano D'Errico[1,2], Nicola Borbone[1,2], Bruno Catalanotti[1], Agnese Secondo[3], Tiziana Petrozziello[3], Ilaria Piccialli[3], Anna Pannaccione[3], Valeria Costantino[1], Luciano Mayol[1], Gennaro Piccialli[1,3] and Giorgia Oliviero[2,4,*]

[1]Dipartimento di Farmacia, Università degli Studi di Napoli Federico II, Via D. Montesano 49, 80131 Napoli, Italy

[2]SYSBIO.IT, Centre of Systems Biology, University of Milano-Bicocca, Milano, Italy

[3]Divisione di Farmacologia, Dipartimento di Neuroscienze, Scienze Riproduttive e Odontostomatologiche, Scuola di Medicina, Università degli Studi di Napoli Federico II, Via S. Pansini 5, 80131 Napoli, Italy

[4]Dipartimento di Medicina Molecolare e Biotecnologie Mediche, Università degli Studi di Napoli Federico II, Via S. Pansini 5, 80131 Napoli, Italy

*Corresponding author: golivier@unina.it

^{1}H- and ^{31}P-NMR spectra of compound **16** S3

^{1}H- and ^{31}P-NMR spectra of compounds **17** S4

^{1}H- and ^{31}P-NMR spectra of compounds **19** S5

^{1}H- and ^{31}P-NMR spectra of compounds **21** S6

^{1}H- and ^{31}P-NMR spectra of compounds **22** S7

^{1}H- and ^{31}P-NMR spectra of compounds **23** S8

^{1}H- and ^{31}P-NMR spectra of compounds **24** S9

^{1}H- and ^{31}P-NMR spectra of compounds **25** S10

^{1}H- and ^{31}P-NMR spectra of compounds **26** S11

^{1}H- and ^{31}P-NMR spectra of compounds **13** S12

^{13}C NMR spectrum of compound **16** S13

^{13}C NMR spectrum of compound **17** S14

^{13}C NMR spectrum of compound **19** S15

^{13}C NMR spectrum of compound **21** S16

^{13}C NMR spectrum of compound **22** S17

^{13}C NMR spectrum of compound **23** S18

^{13}C NMR spectrum of compound **24** S19

^{13}C NMR spectrum of compound **25** S20

^{13}C NMR spectrum of compound **26** S21

Table S1 S22

Figure S1 S22

Table S2 S23

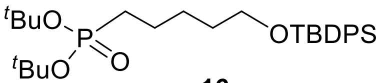

16

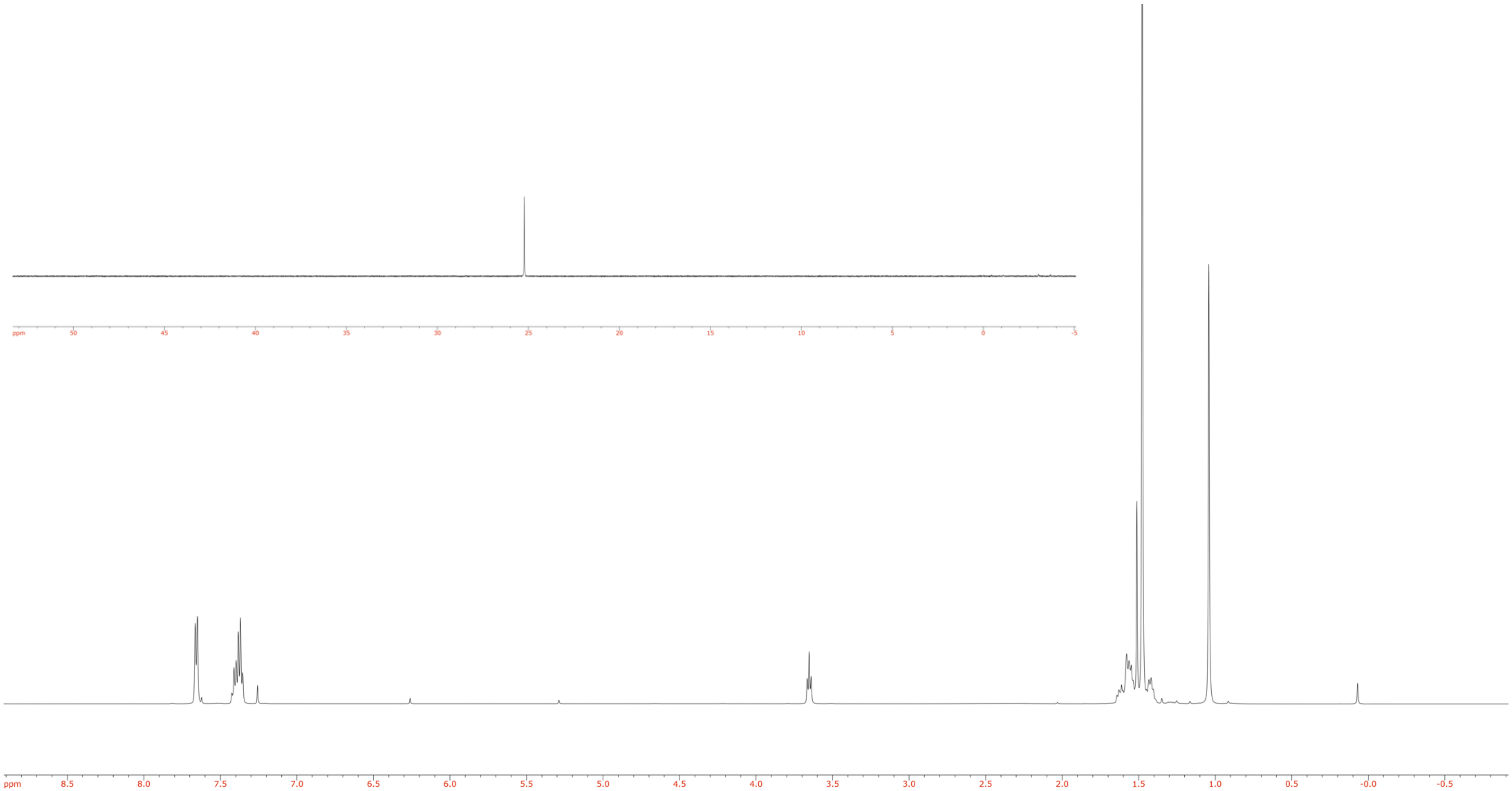
ppm 50 45 40 35 30 25 20 15 10 5 0 -5
ppm 8.5 8.0 7.5 7.0 6.5 6.0 5.5 5.0 4.5 4.0 3.5 3.0 2.5 2.0 1.5 1.0 0.5 -0.0 -0.5

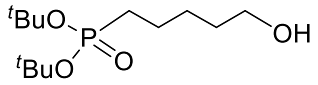

17

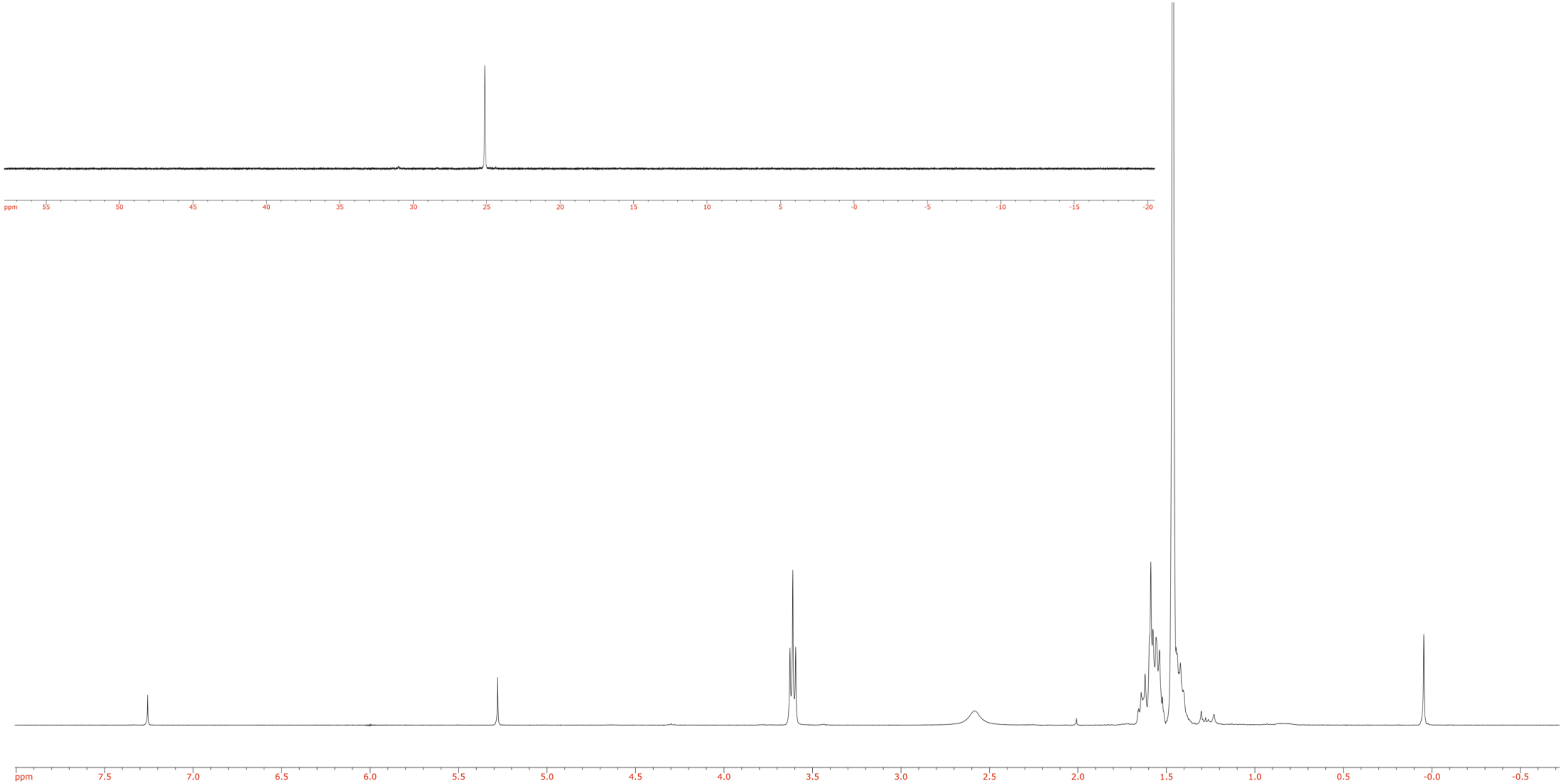

ppm 55 50 45 40 35 30 25 20 15 10 5 -0 -5 -10 -15 -20
ppm 7.5 7.0 6.5 6.0 5.5 5.0 4.5 4.0 3.5 3.0 2.5 2.0 1.5 1.0 0.5 -0.0 -0.5

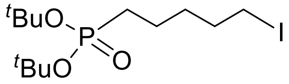

^{t}BuO
P
O
^{t}BuO
I

19

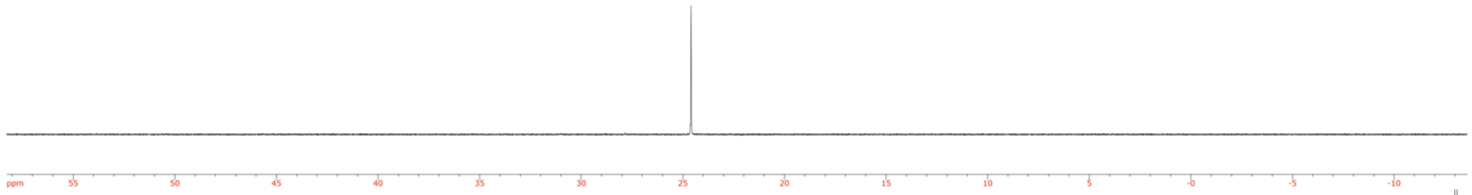

ppm
55
50
45
40
35
30
25
20
15
10
5
-0
-5
-10

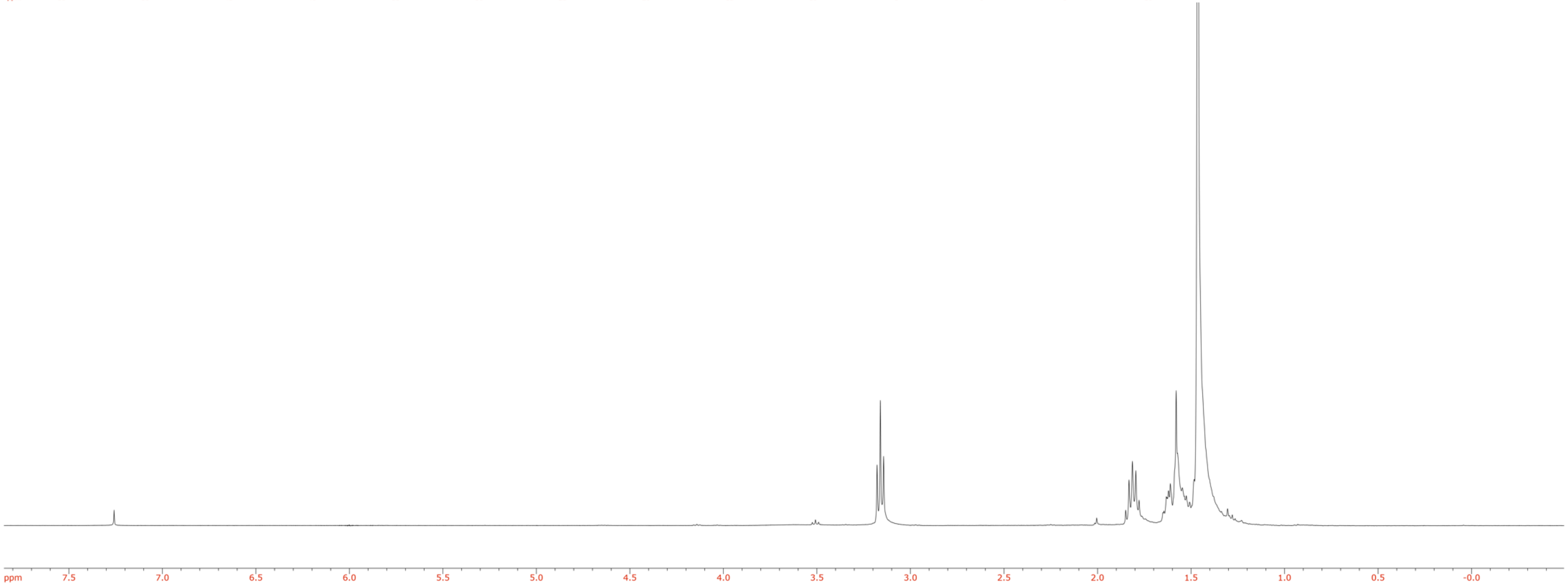

ppm
7.5
7.0
6.5
6.0
5.5
5.0
4.5
4.0
3.5
3.0
2.5
2.0
1.5
1.0
0.5
-0.0

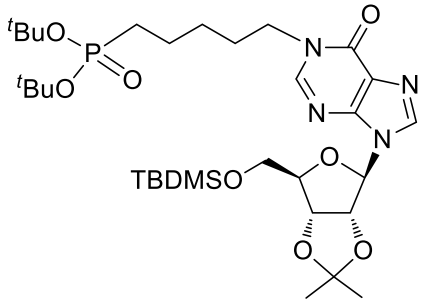
tBuO
tBuO
TBDMSO

21

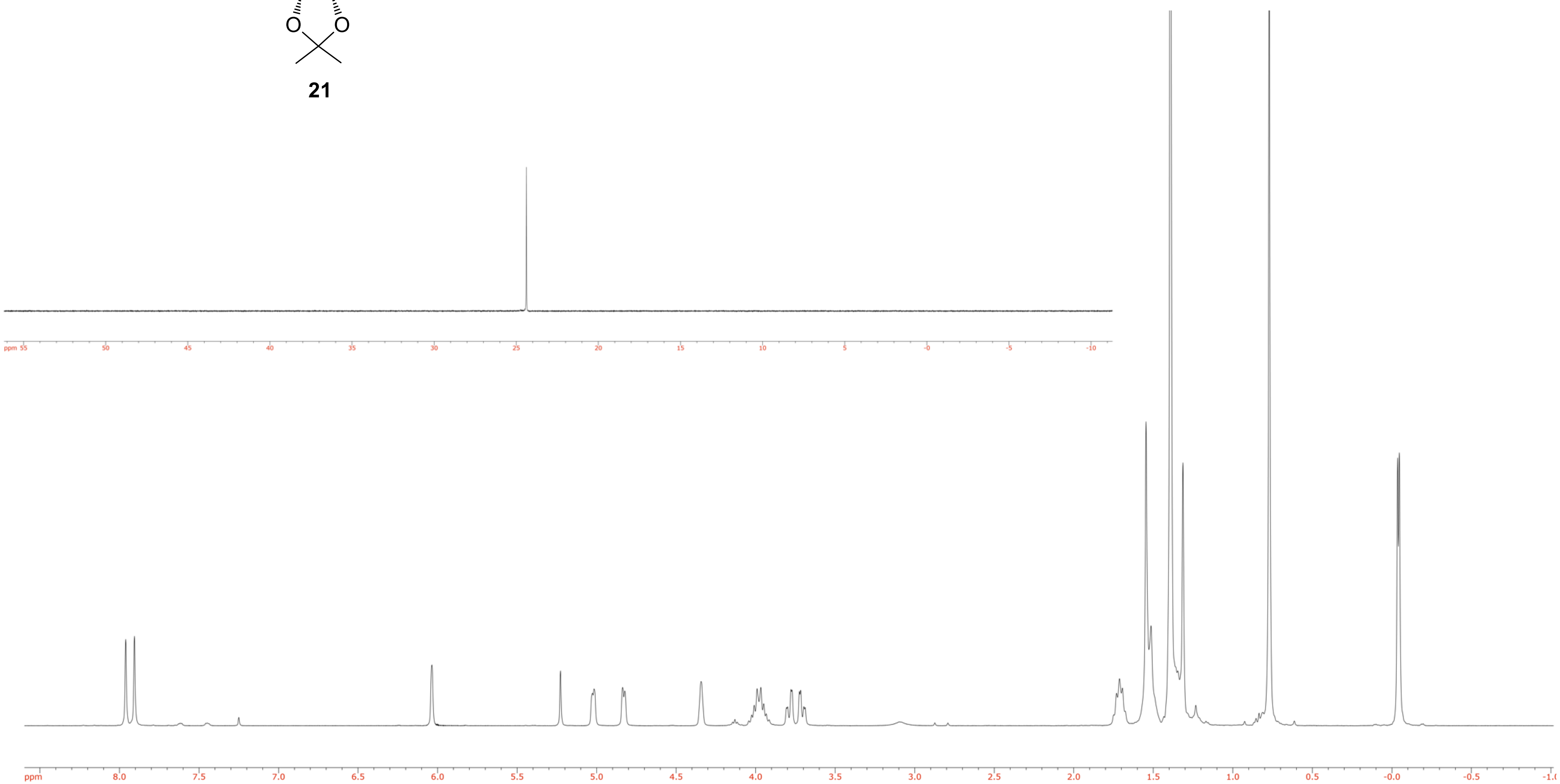
ppm 55 50 45 40 35 30 25 20 15 10 5 -0 -5 -10
ppm 8.0 7.5 7.0 6.5 6.0 5.5 5.0 4.5 4.0 3.5 3.0 2.5 2.0 1.5 1.0 0.5 -0.0 -0.5

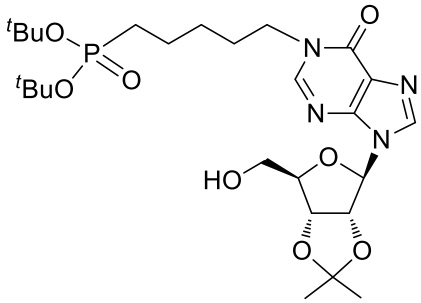

tBuO
tBuO
P
O
N
O
N
N
N
N
HO
O
O
O

22

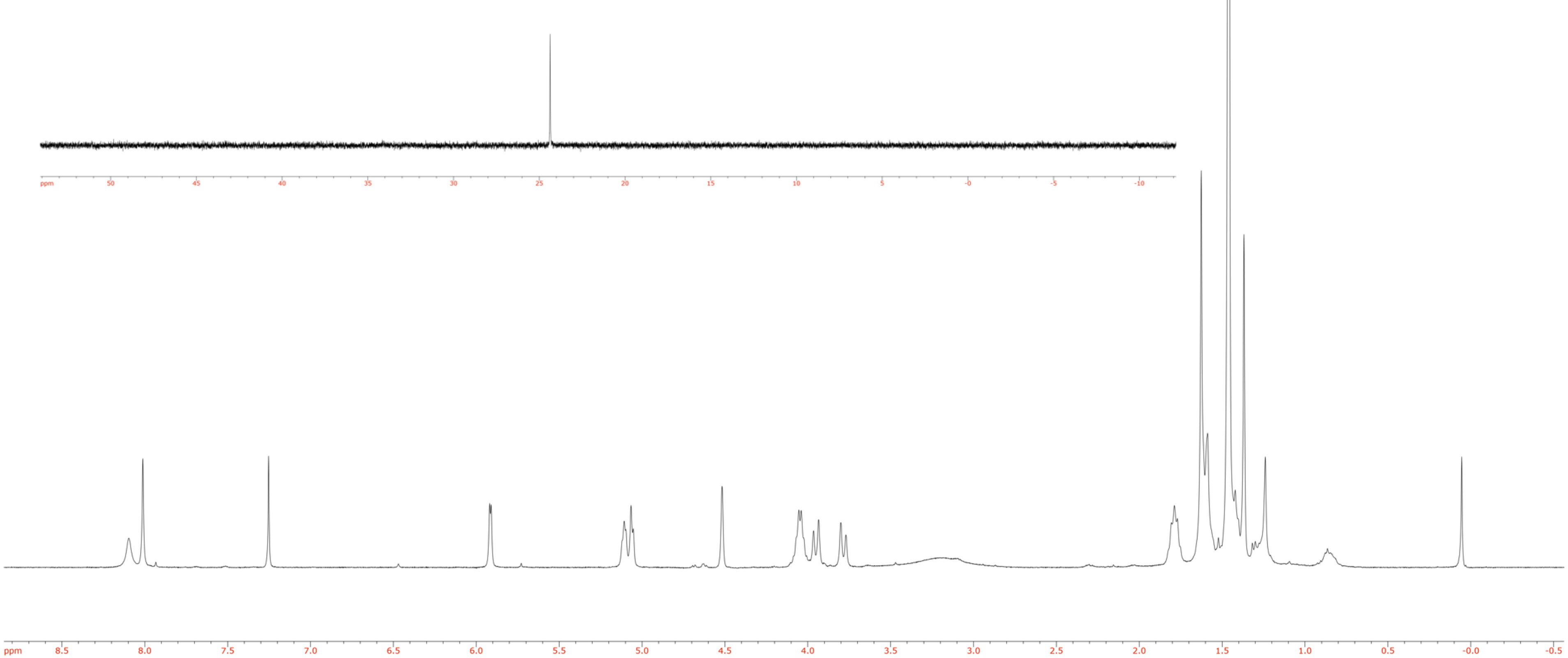

ppm 50 45 40 35 30 25 20 15 10 5 0 -5 -10
ppm 8.5 8.0 7.5 7.0 6.5 6.0 5.5 5.0 4.5 4.0 3.5 3.0 2.5 2.0 1.5 1.0 0.5 -0.0 -0.5

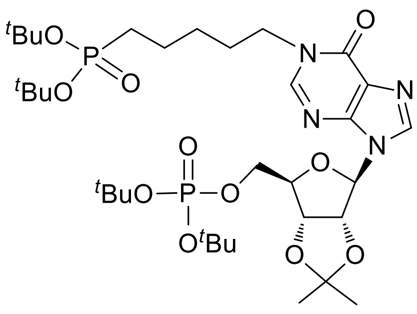
tBuO
tBuO
O
N
N
N
N
O
tBuO
OtBu
O
O
O
O

23

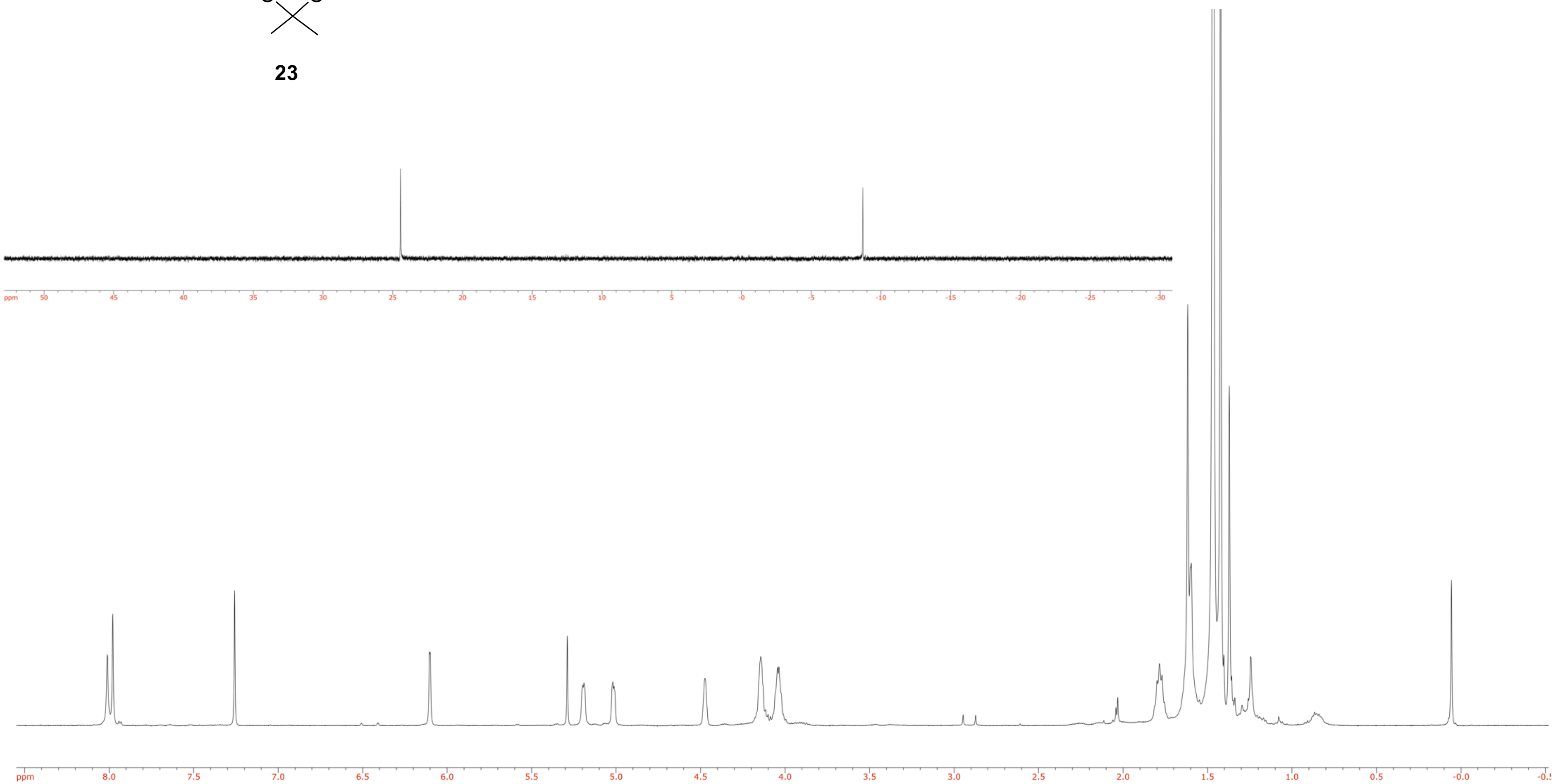
ppm
50
45
40
35
30
25
20
15
10
5
-0
-5
-10
-15
-20
-25
-30
ppm
8.0
7.5
7.0
6.5
6.0
5.5
5.0
4.5
4.0
3.5
3.0
2.5
2.0
1.5
1.0
0.5
-0.0

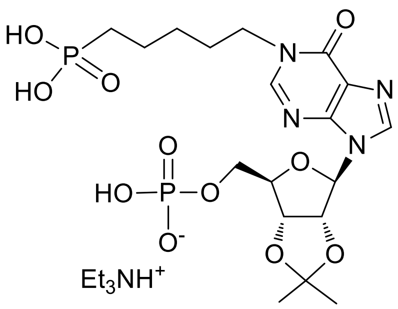

24

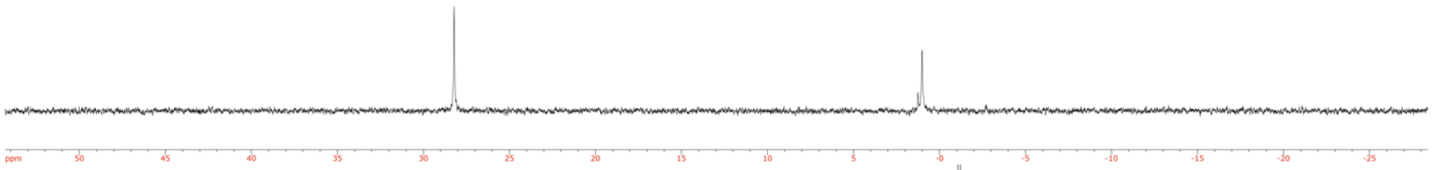

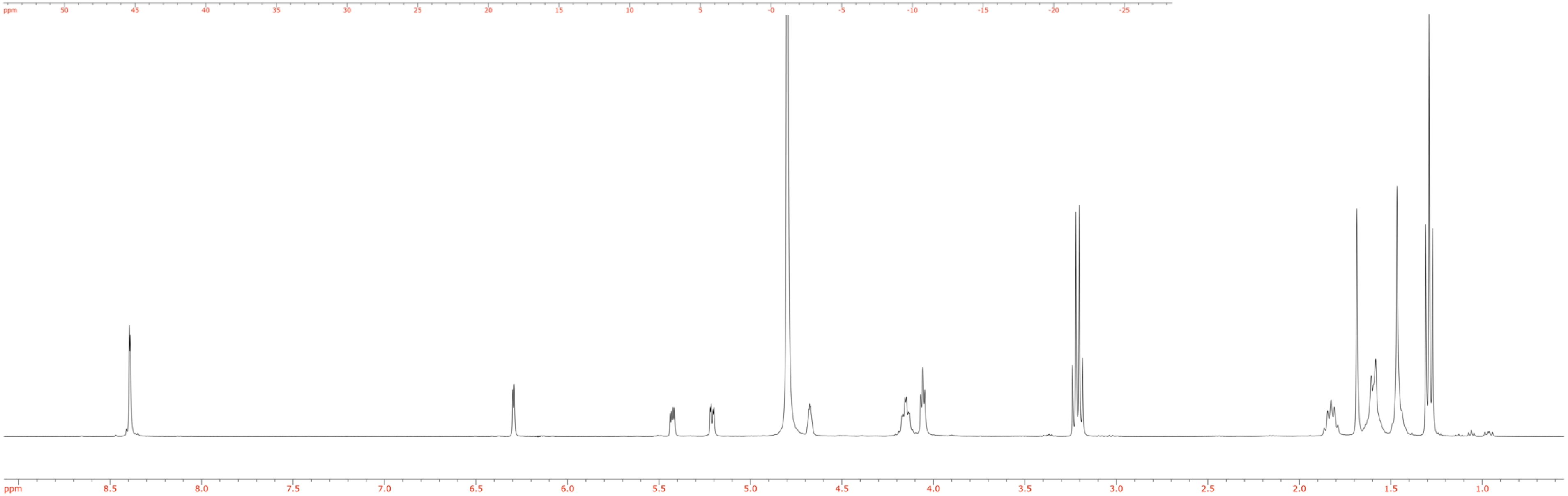

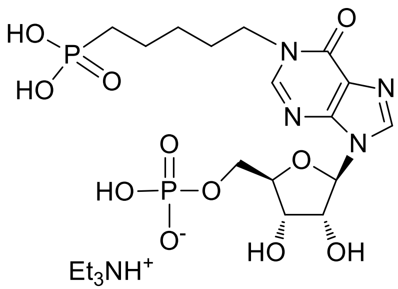

25

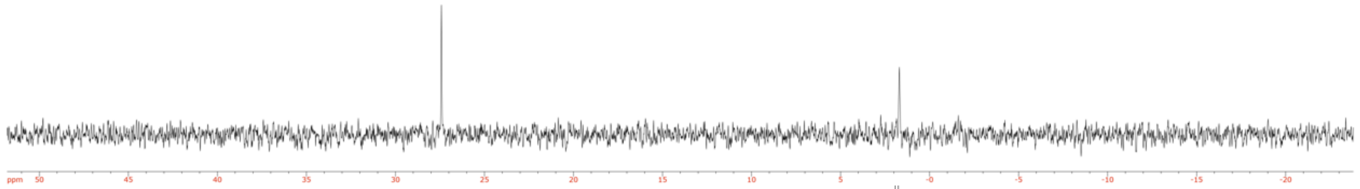
ppm 50 45 40 35 30 25 20 15 10 5 0 -5 -10 -15 -20

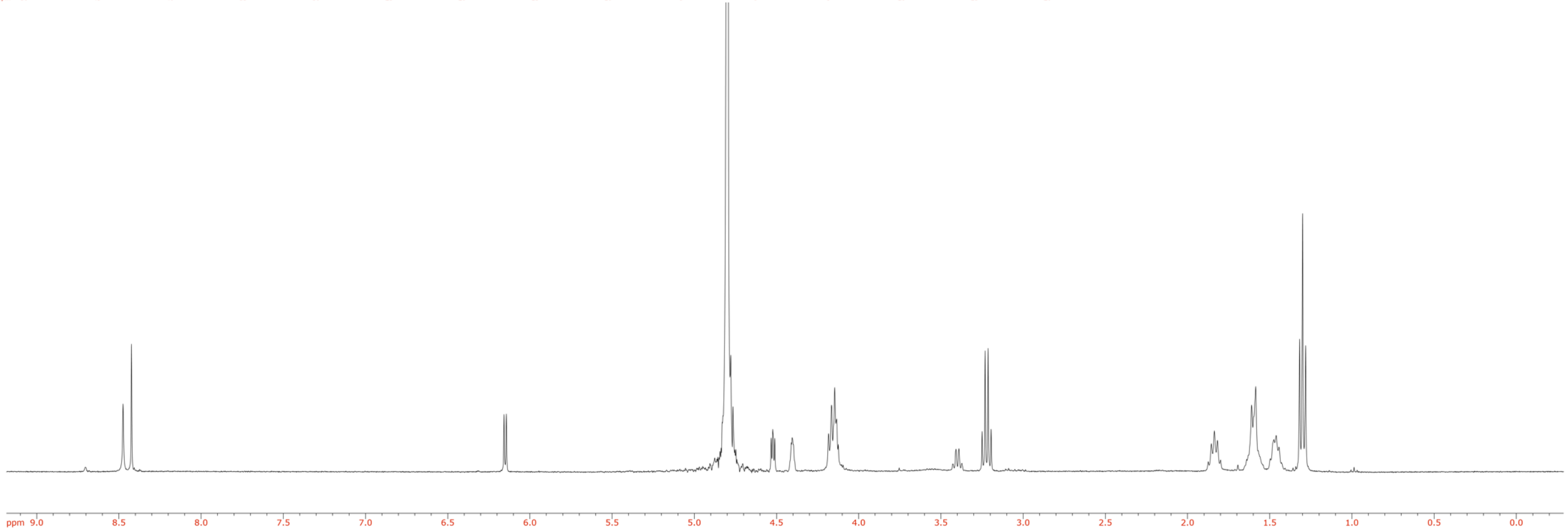
ppm 9.0 8.5 8.0 7.5 7.0 6.5 6.0 5.5 5.0 4.5 4.0 3.5 3.0 2.5 2.0 1.5 1.0 0.5 0.0

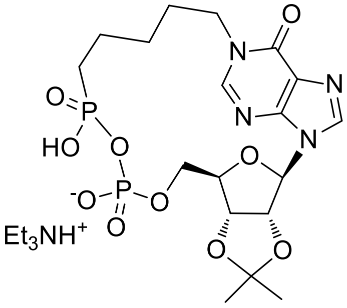

26

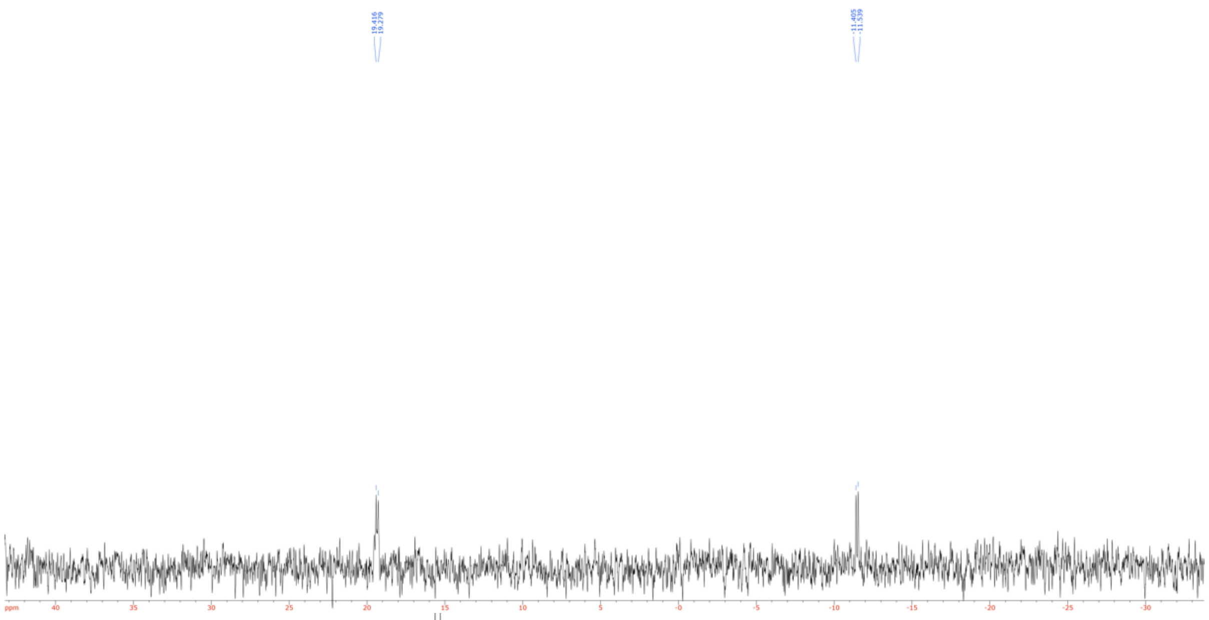

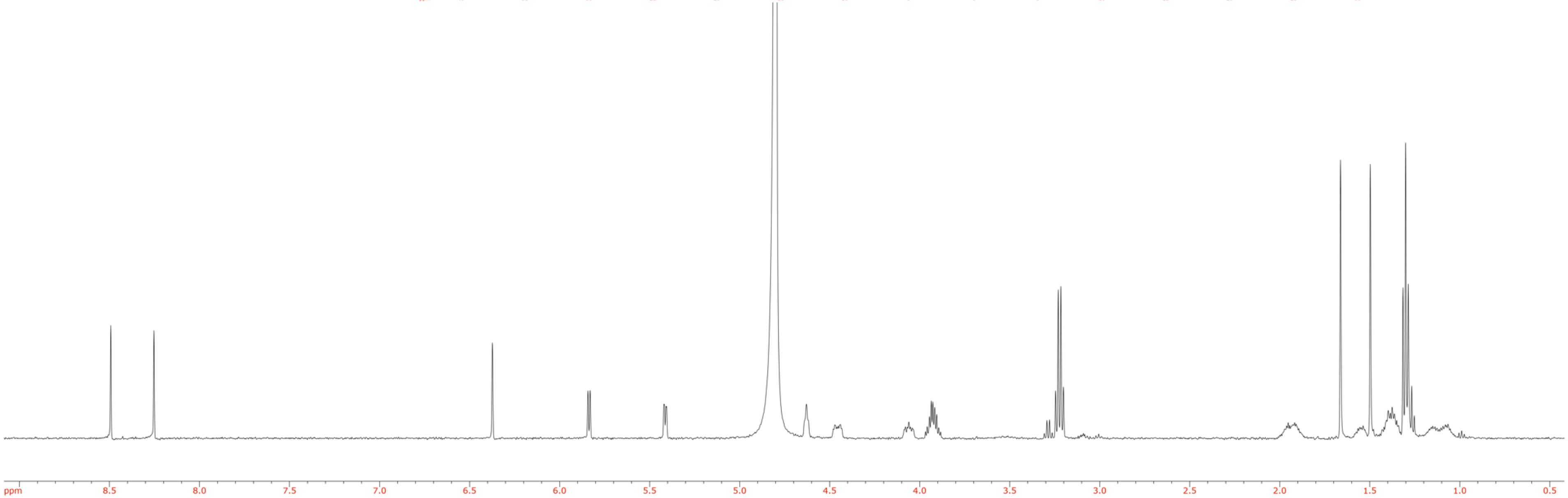

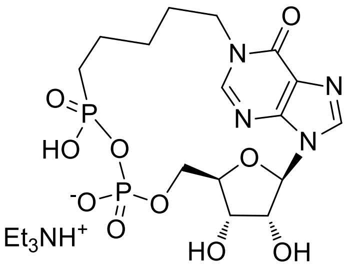

O
N
N
N
N
O
P
HO
O
O
O
P
O
Et_3NH^+
O
HO
OH

13

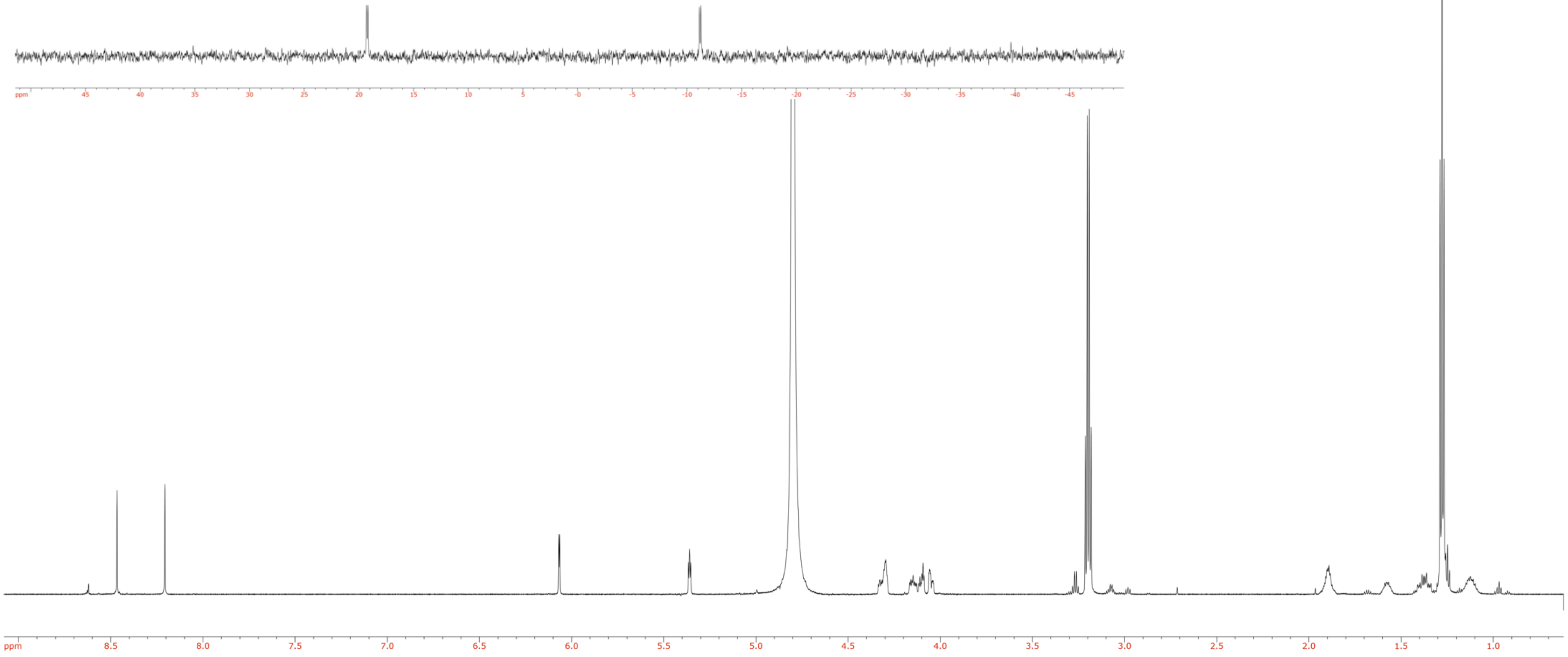

ppm
8.5
8.0
7.5
7.0
6.5
6.0
5.5
5.0
4.5
4.0
3.5
3.0
2.5
2.0
1.5
1.0

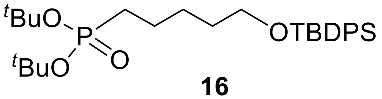

16

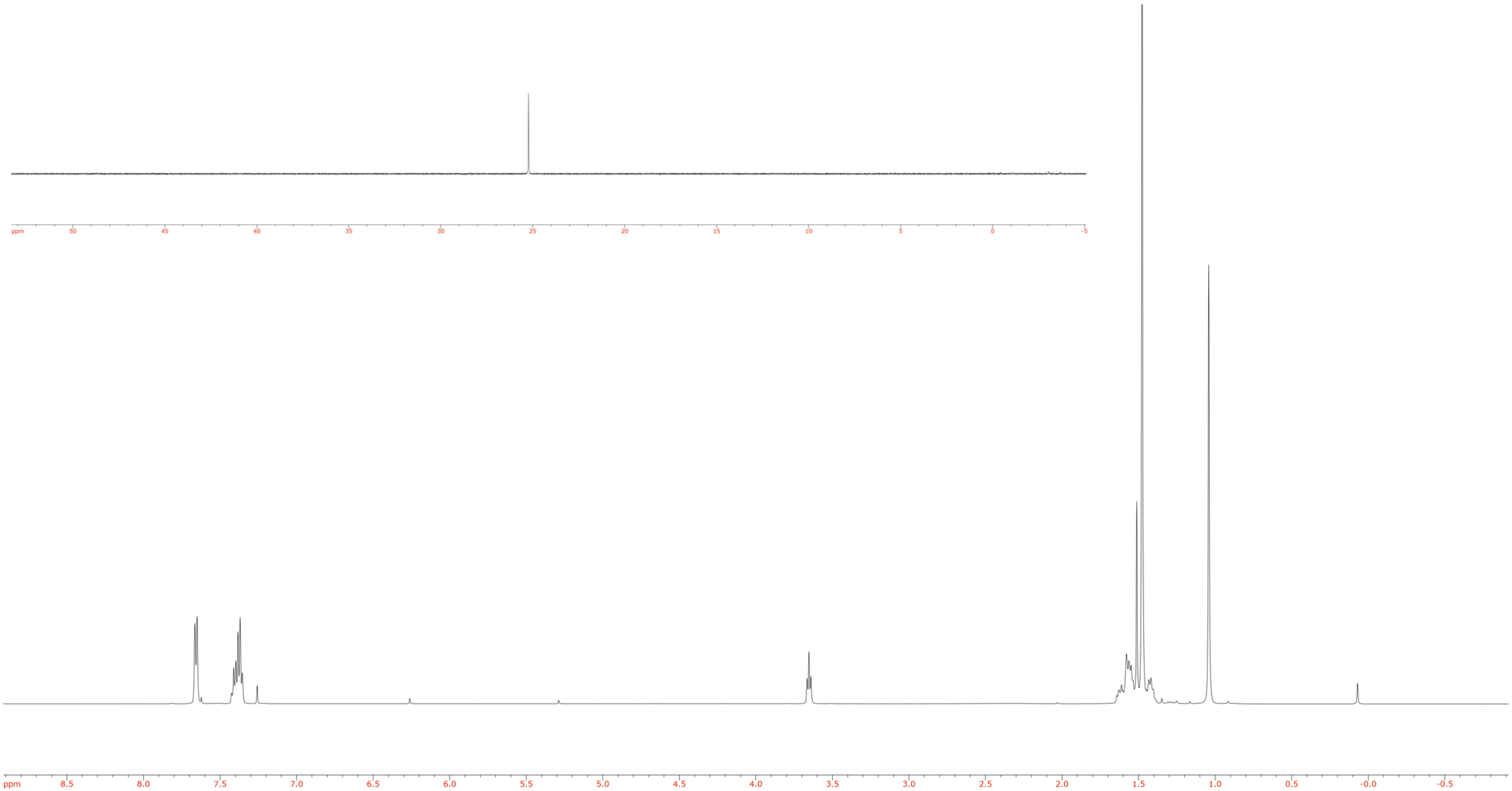

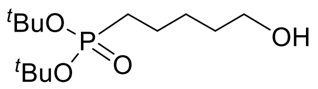

17

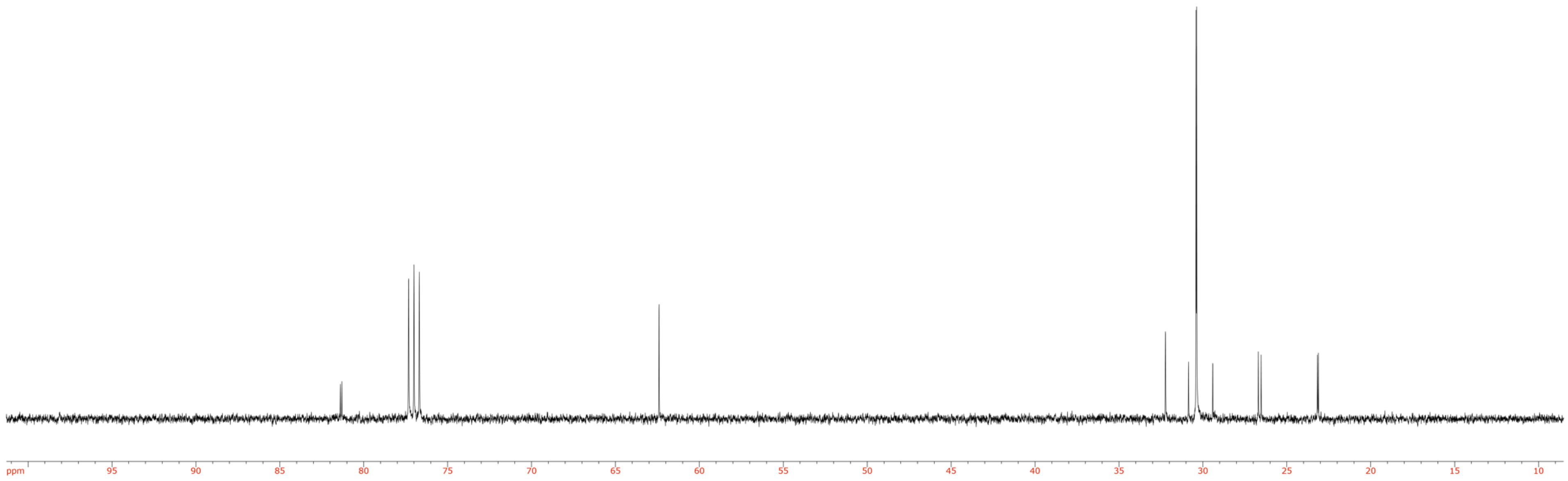

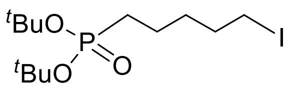

tBuO
tBuO
P
O
I

19

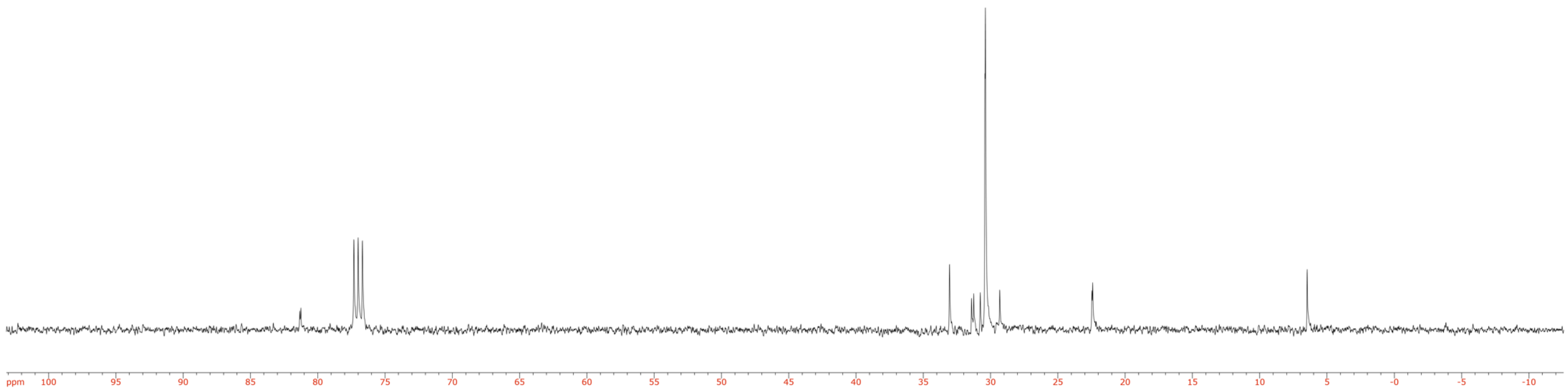

ppm
100
95
90
85
80
75
70
65
60
55
50
45
40
35
30
25
20
15
10
5
-0
-5
-10

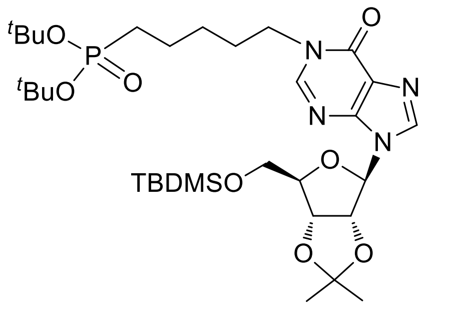

tBuO
P
O
tBuO
N
O
N
N
N
TBDMSO
O
O
O

21

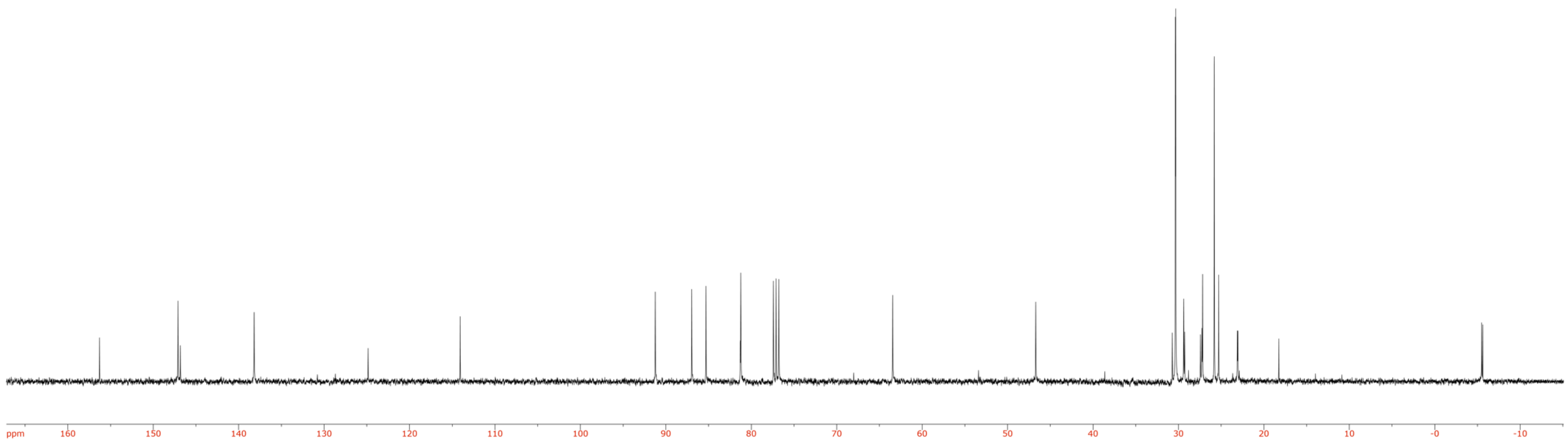

ppm
160
150
140
130
120
110
100
90
80
70
60
50
40
30
20
10
-0
-10

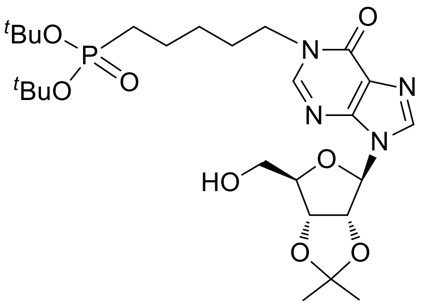

^{t}BuO
^{t}BuO
P
O
N
O
N
N
N
HO
O
O
O

22

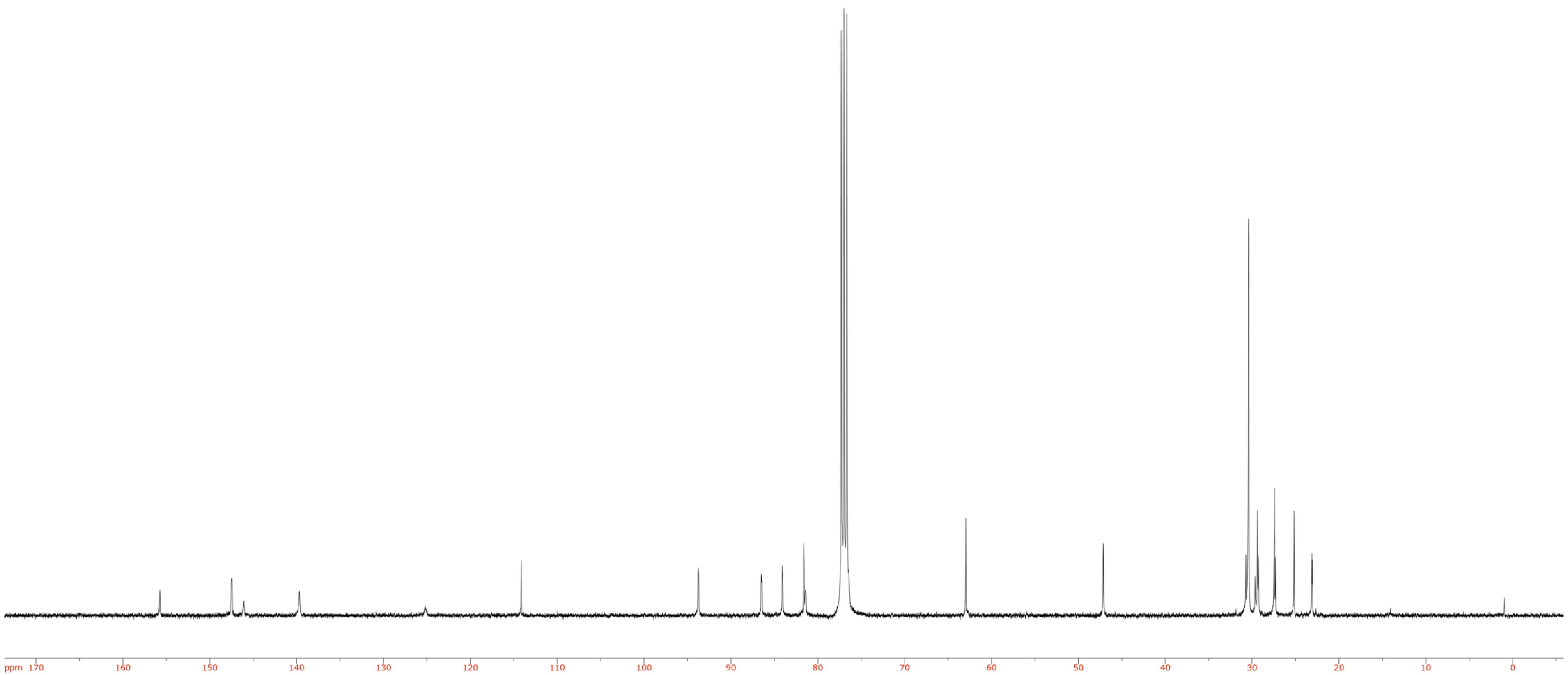

ppm 170 160 150 140 130 120 110 100 90 80 70 60 50 40 30 20 10 0

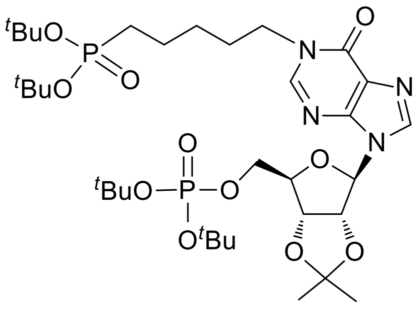
tBuO
tBuO
O
N
N
N
N
O
O
tBuO
OtBu
O
O
O
P
P

23

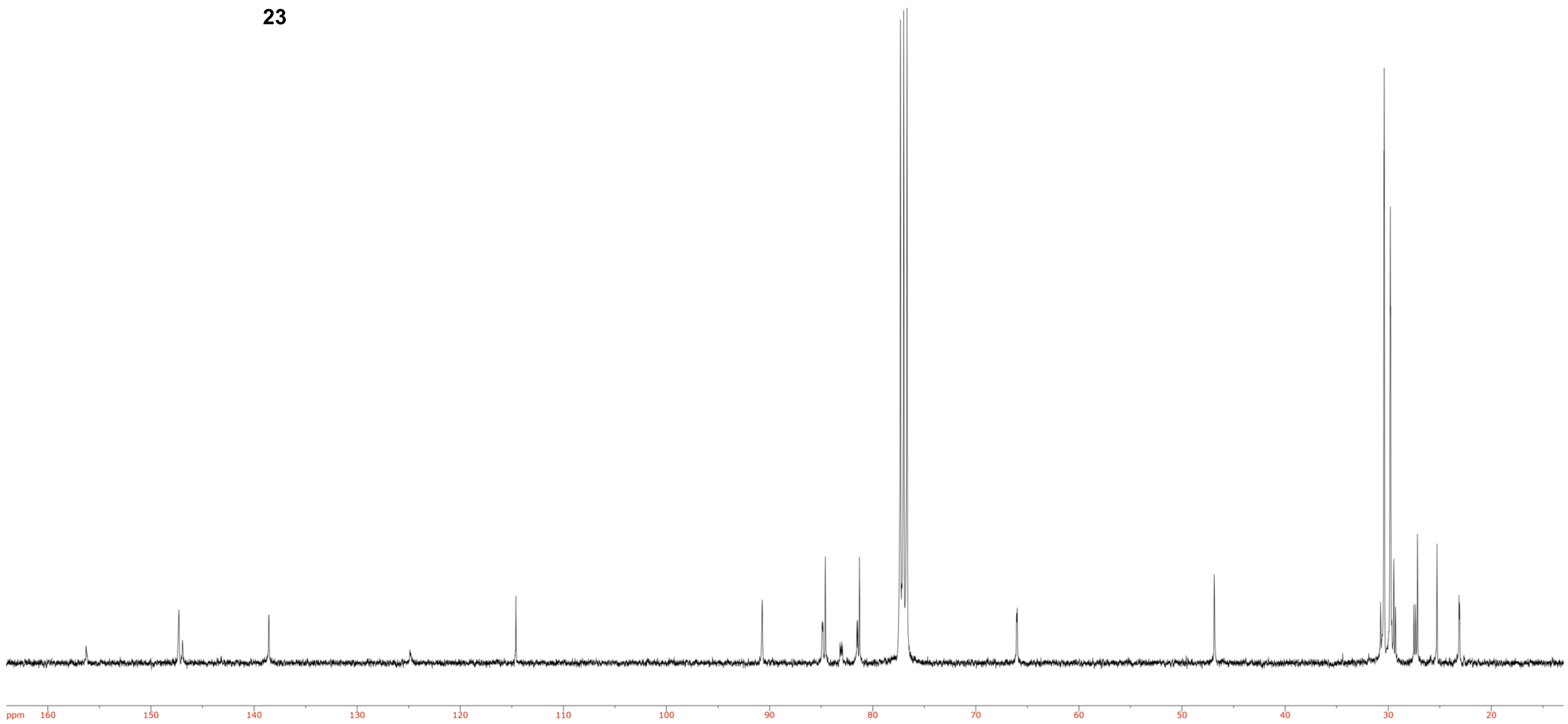
ppm 160 150 140 130 120 110 100 90 80 70 60 50 40 30 20

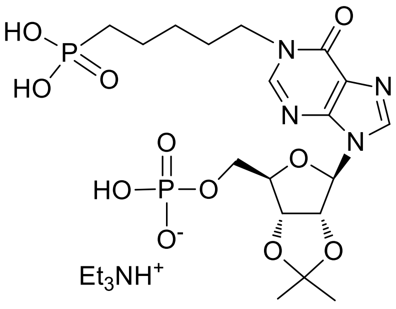
HO
P
O
HO
N
O
N
N
N
O
HO
P
O
O
O
N
O
O
O-
Et3NH+

24

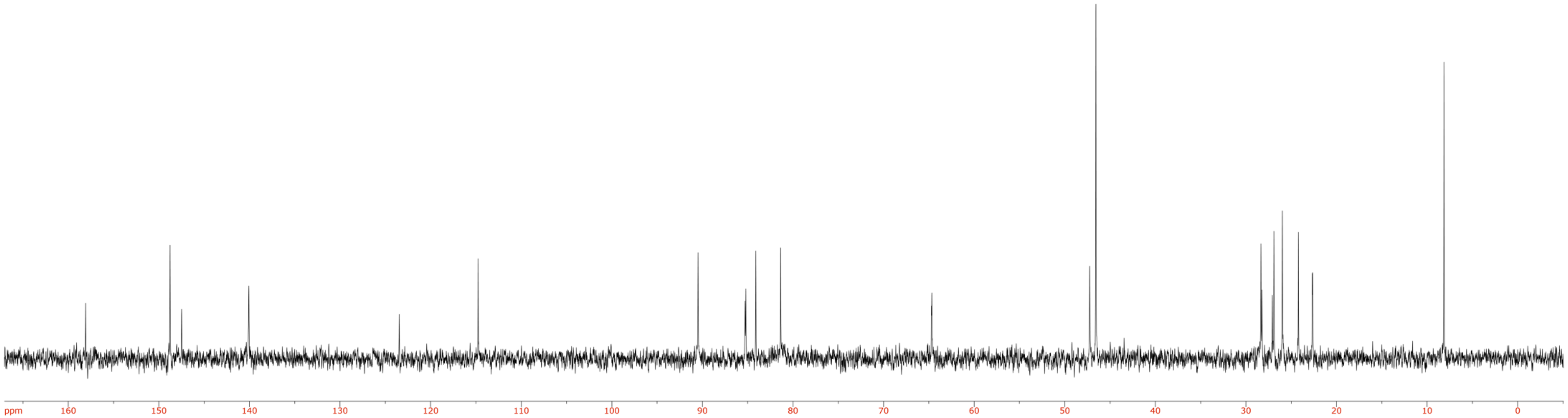
ppm
160
150
140
130
120
110
100
90
80
70
60
50
40
30
20
10
0

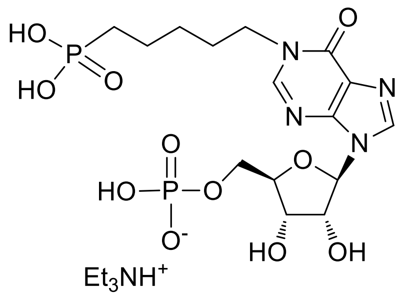
HO
HO
P
O
N
O
N
N
N
O
HO
P
O
O
O-
Et3NH+
HO
OH

25

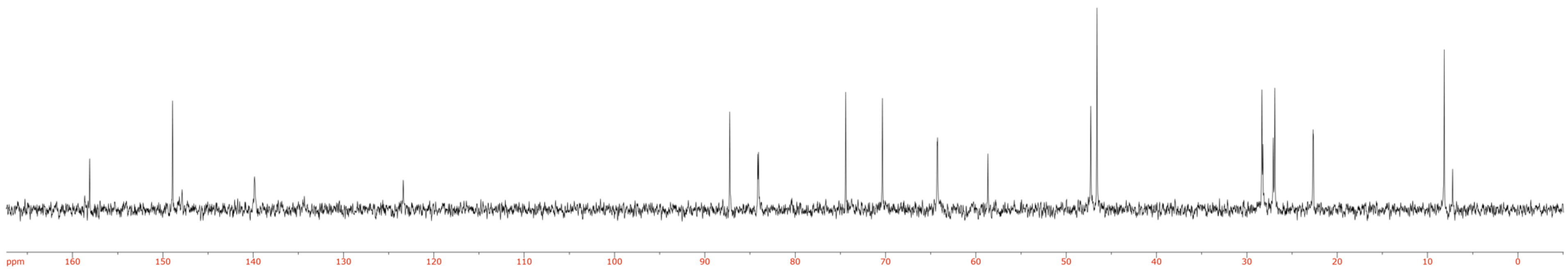
ppm
160
150
140
130
120
110
100
90
80
70
60
50
40
30
20
10
0

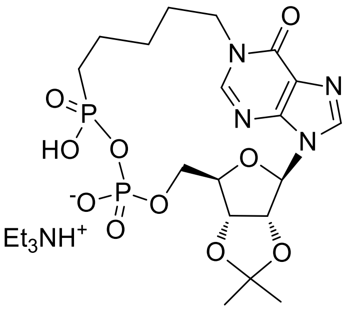

26

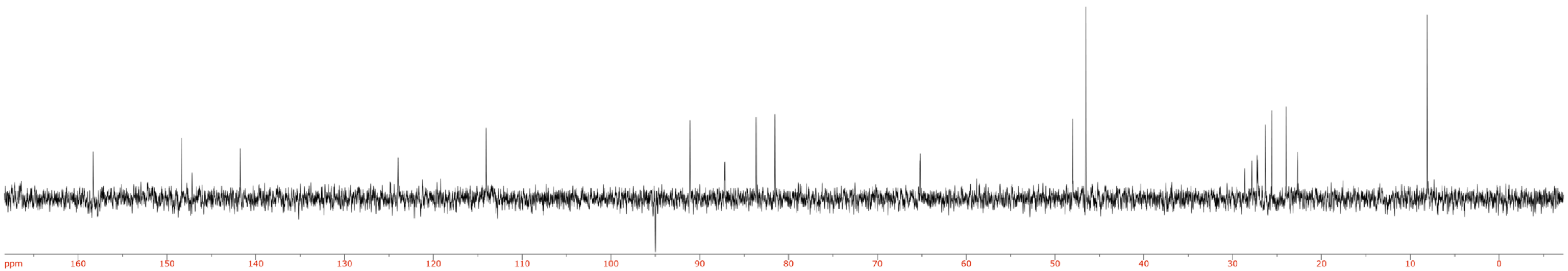

Table S1. Lipophilicity, Relative OPLS 2005 energies and geometrical features of conformers within 5 Kcal/mol from the lowest energy conformation. Atoms used to calculate geometric features are described in Figure 3 of the main text.

Cmpd	AlogP	ΔE (kcal/mol)	d_1 (Å)	d_2 (Å)	d_2 (Å)	θ_1 (°)	θ_2 (°)	θ_3 (°)	θ_4 (°)
cpIPP (**13**)	-2.4	0.0	8,4	5,6	4,7	73	-38	-54	-140
		4.4	8,2	5,4	6,2	74	67	-56	-113
cbIDP (**10**)	-2.5	0.0	7,2	4,0	3,9	68	-177	-178	-81
		3.6	7,8	5,4	5,8	65	169	-171	63
		4.4	6,8	4,7	5,9	177	-176	57	-63
		4.7	8,2	5,2	4,7	-46	-54	62	-78
		4.8	7,6	4,2	4,6	69	171	63	80
		4.9	6,6	5,2	4,2	-89	-157	-40	59
cpIDP (**11**)	-2.0	0	8,9	5,6	5,1	-33	-62	54	51
		4.2	6,5	4,8	4,1	171	-39	79	38
		4.3	9,0	5,5	5,2	-12	-34	85	42
		4.8	8,1	6,2	6,6	-81	-69	50	52

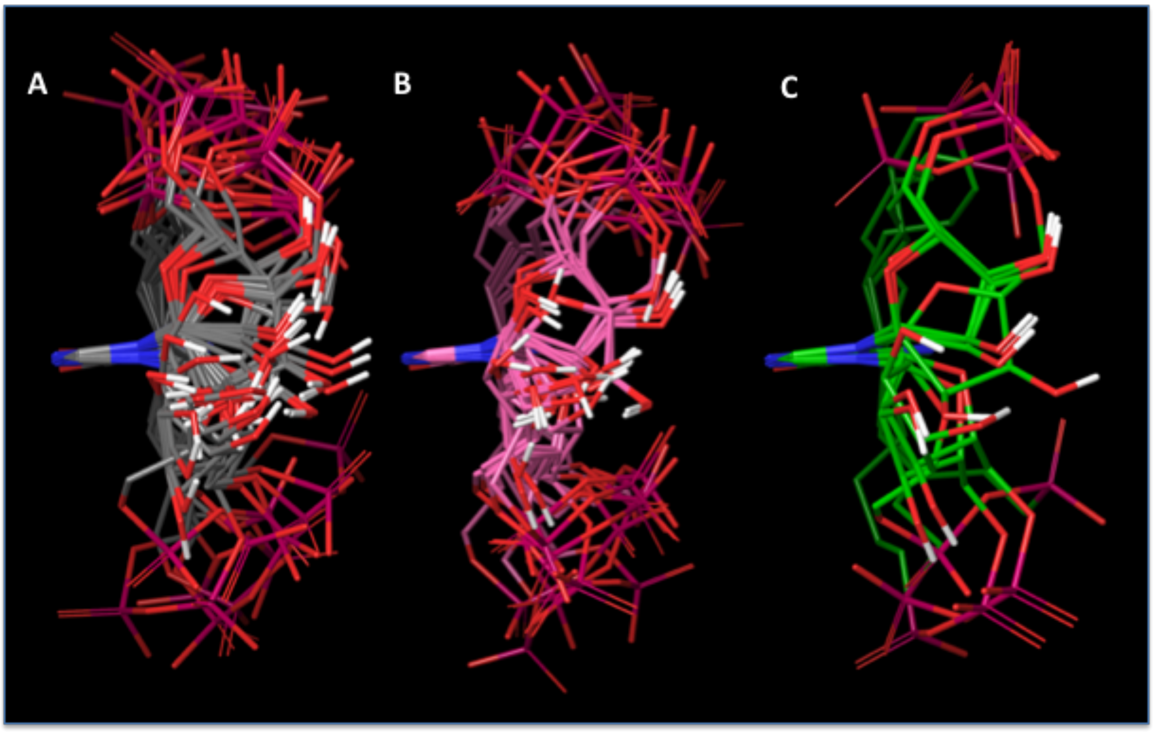

Figure S1. Superimposition on the inosine ring of conformers within 10 kcal/mol from global minimum: A) cbIDP (**10**, grey), B) cpIDP (**11**, pink), and C) cpIPP (**13**, green). Non-polar hydrogens were omitted for sake of clarity. Oxygens were reported in red, nitrogens in blue, phosphates in magenta, hydrogens in white.

Table S2. Lipophilicity, Relative OPLS 2005 energies and geometrical features of conformers within 10 Kcal/mol from the lowest energy conformation. Atoms used to calculate geometric features are described in Figure 3 of the main text.

Cmpd	AlogP	ΔE (kcal/mol)	d_1 (Å)	d_2 (Å)	d_2 (Å)	θ_1 (°)	θ_2 (°)	θ_3 (°)	θ_4 (°)
cpIPP (**13**)	-2.4	0.0	8,4	5,6	4,7	73	-38	-54	-140
		4.4	8,2	5,4	6,2	74	67	-56	-113
		5.8	8,4	5,8	5,7	82	-41	72	179
		6.9	8,4	5,9	5,6	65	-40	-67	-158
		9.5	8,1	6,0	6,6	68	46	60	-172
		10.0	8,0	5,0	4,4	62	-173	62	171
cbIDP (**10**)	-2,5	0	7,2	4,0	3,9	68	-177	-178	-81
		3,6	7,8	5,4	5,8	65	169	-171	63
		4,4	6,8	4,7	5,9	177	-176	57	-63
		4,7	8,2	5,2	4,7	-46	-54	62	-78
		4,8	7,6	4,2	4,6	69	171	63	80
		4,9	6,6	5,2	4,2	-89	-157	-40	59
		5,8	7,9	6,1	6,2	73	-53	69	74
		6,5	7,3	4,9	6,8	81	75	168	73
		7,2	6,8	3,6	3,9	-61	-169	166	-84
		7,3	7,9	5,0	4,2	-40	175	58	-86
		7,5	6,3	5,0	5,8	-59	-68	-76	58
		7,8	8,2	5,4	4,5	71	-59	62	77
		8,4	7,0	4,7	6,4	61	164	58	-77
		8,6	7,3	4,6	6,5	89	-55	173	73
		8,7	7,2	4,1	5,5	-74	179	-64	77
		8,9	7,9	6,1	7,0	79	56	52	-78
		9,1	7,4	4,0	4,5	66	164	-67	77
		9,2	7,6	4,4	4,8	167	50	-60	-72
		9,4	7,6	6,1	6,8	64	58	-171	79
		9,5	8,2	5,2	4,6	-46	-53	165	-78
		9,6	7,7	5,5	6,4	71	153	171	60
		9,9	7,2	5,8	6,6	62	167	60	-68
		10,0	7,4	4,7	4,6	-61	63	-159	67

cpIDP (**11**)	-2.0	0,0	8,9	5,6	5,1	-33	-62	54	51
		4,2	6,5	4,8	4,1	171	-39	79	38
		4,3	9,0	5,5	5,2	-12	-34	85	42
		4,8	8,1	6,2	6,6	-81	-69	50	52
		5,5	8,6	5,9	5,8	-25	-59	57	49
		5,7	8,4	6,4	7,7	89	-168	-53	60
		5,9	8,2	5,0	4,7	-162	-66	49	36
		6,5	8,7	5,8	6,5	68	42	-74	-44
		6,9	7,7	5,7	4,2	-160	-54	64	34
		7,9	8,8	5,5	5,5	175	70	-176	37
		8,5	7,2	4,5	6,4	59	152	35	-55
		9,0	7,8	5,8	4,6	-111	159	44	-52
		9,2	6,4	4,9	4,6	154	174	58	-35
		9,3	7,3	3,9	4,3	-164	172	58	-35
		9,5	8,9	5,3	5,2	81	-168	-54	61
		9,7	8,4	6,1	6,8	86	65	-54	-51
		9,9	7,4	5,0	6,6	-23	-55	61	48
